# Grassroots and Youth-Led Climate Solutions From The Gambia

**DOI:** 10.3389/fpubh.2022.784915

**Published:** 2022-04-07

**Authors:** Ana Bonell, Jainaba Badjie, Sariba Jammeh, Zakari Ali, Muhammed Hydara, Adesina Davies, Momodou Faal, Aliyu Nuhu Ahmed, William Hand, Andrew M. Prentice, Kris A. Murray, Pauline Scheelbeek

**Affiliations:** ^1^Medical Research Council Unit The Gambia at London School of Hygiene and Tropical Medicine, Fajara, The Gambia; ^2^Centre on Climate Change and Planetary Health, London School of Hygiene and Tropical Medicine, London, United Kingdom; ^3^Great Institute, Bijilo, The Gambia; ^4^Banjul American International School, Fajara, The Gambia; ^5^Medical Research Council (MRC) Centre for Global Infectious Disease Analysis, School of Public Health, Imperial College London, London, United Kingdom

**Keywords:** climate change, solutions, youth, West Africa, public engagement

## Abstract

Climate change and environmental degradation are among the greatest threats to human health. Youth campaigners have very effectively focused global attention on the crisis, however children from the Global South are often under-represented (sometimes deliberately) in the dialogue. In The Gambia, West Africa, the impacts of climate change are already being directly experienced by the population, and this will worsen in coming years. There is strong government and community commitment to adapt to these challenges, as evidenced by The Gambia currently being the only country on target to meet the Paris agreement according to the Nationally Determined Contributions, but again children's voices are often missing—while their views could yield valuable additional insights. Here, we describe a “Climate Change Solutions Festival” that targeted and engaged school children from 13 to 18 years, and is to our knowledge, the first peer-to-peer (and student-to-professional) learning festival on climate change solutions for students in The Gambia. The event gave a unique insight into perceived climate change problems and scalable, affordable and sometimes very creative solutions that could be implemented in the local area. Logistical and practical methods for running the festival are shared, as well as details on all solutions demonstrated in enough detail to be duplicated. We also performed a narrative review of the most popular stalls to explore the scientific basis of these solutions and discuss these in a global context. Overall, we find extremely strong, grass-roots and student engagement in the Gambia and clear evidence of learning about climate change and the impacts of environmental degradation more broadly. Nevertheless, we reflect that in order to enact these proposed local solutions further steps to evaluate acceptability of adoption, feasibility within the communities, cost-benefit analyses and ability to scale solutions are needed. This could be the focus of future experiential learning activities with students and partnering stakeholders.

## Introduction

Humanity's impact on the environment including the climate has been acknowledged as one of the greatest threats to human health (1, 2). In their latest report IPCC state that it is “*unequivocal that human influence has warmed the atmosphere, ocean and land,”* with further evidence that this has led to increased frequency of weather and climate extremes, including heatwaves, heavy precipitation, droughts, and tropical cyclones (3). This, in turn, has already had a devastating effect on biodiversity, food security, and livelihoods in various parts of the world (4, 5).

Children, now and in the future, will be the ones to bear the burden of humanity's environmental impacts, both in terms of direct impacts on their health but also in a multitude of indirect effects ranging from food security to economic impacts (6). Youth representatives from around the world have forced climate change into the global agenda where they have campaigned to highlight the need for immediate action (7). The passion and power of this group is inspirational but also demonstrates the need to engage with the youth in finding solutions and shaping the future in line with their visions (8). Nonetheless, children's voices are often not acknowledged or considered important in environmental policy settings (9).

Additionally, accessing school children in many areas of the world, including The Gambia has its own challenges. The global population has become increasingly connected, however, social media, email and internet access to connect and communicate remains uncommon in many areas and/or among certain societal groups, especially in rural Sub-Saharan Africa, adding to logistical difficulties in this endeavor (10, 11).

In order to encourage youth civic engagement, it has been shown that influences from both home and the school environment are important (12, 13). By exposing students to the ideas, problems and solutions to climate change, focus and awareness can be garnered around the issues, empowering discussions and allowing students to drive the conversation. Interactive and peer-to-peer learning methods have been shown to be effective at delivery of an educational message and to be both engaging and motivating (14, 15).

Here, we present an interactive, peer-to-peer (and student-to-professional) learning “Climate Change Solutions Festival,” targeting school children aged 13-18 years old nationwide. This work was informed by a previous public engagement activity in January 2020 at Bakau Newtown Primary School, Fajara, The Gambia, which gave insights into the perceptions of climate change and students' ideas of how to mitigate/adapt to climate change. During this preliminary work, there was a keen interest in the subject, and a wish to share knowledge. We therefore built on this experience with a larger, nationwide festival. The aim of the festival was to learn with, and from, school aged children, non-government organisations (NGOs) and other actors across The Gambia about existing climate change impacts and adaptation/mitigation solutions, to encourage young people to engage in climate and health science, and stimulate discussion among young people and other delegates to consider priorities related to climate change.

Below we provide an overview of this experiential learning event, with details on its inception, design and our approach to channeling these ideas into practical demonstrations run by students and NGOs for students. We also comment on the feasibility or feasibility gaps in scaling up locally proposed solutions to help solve local environmental challenges as gleaned from solution-specific literature review (which is for the most part inaccessible to students prior to designing their solutions), and where they lie in the global perspective.

## Context

West Africa is considered a particularly vulnerable region to the past and projected impacts of climate change (16, 17). The population in this region is already exposed to the impacts of climate change (increased drought, reduced growing season, extreme heat and increase in flooding) and are addressing this by strong government commitments in conjunction with community measures (18). However, the disconnect between resource availability for individuals to adapt to exposures such as extreme heat (e.g., construction materials, digital technology, sustainable, and affordable energy resources) and the increasing impact of climate change on everyday life is nowhere more evident than in The Gambia, the smallest and one of the least developed countries in Sub-Saharan Africa (19). This is recognized by the Gambian government, which is showing global leadership in its climate commitments that align emissions to within a 1.5°C global warming target (18). However, while climate change is currently taught in Gambian Schools, the Ministry of Education are looking to expand the curriculum (20). Accessing schools throughout the country (despite challenges) was important for equity of opportunity, since there are 137 government run schools in the six regions, of which 68% are in region 1 and 2. In addition, the standard teacher-led approach to education means school children learn predominantly only theory which has been shown to lack engagement compared to child-centered approaches (21).

## Programmatic Detail

### Conceptualization and Planning

**Figure 1:**
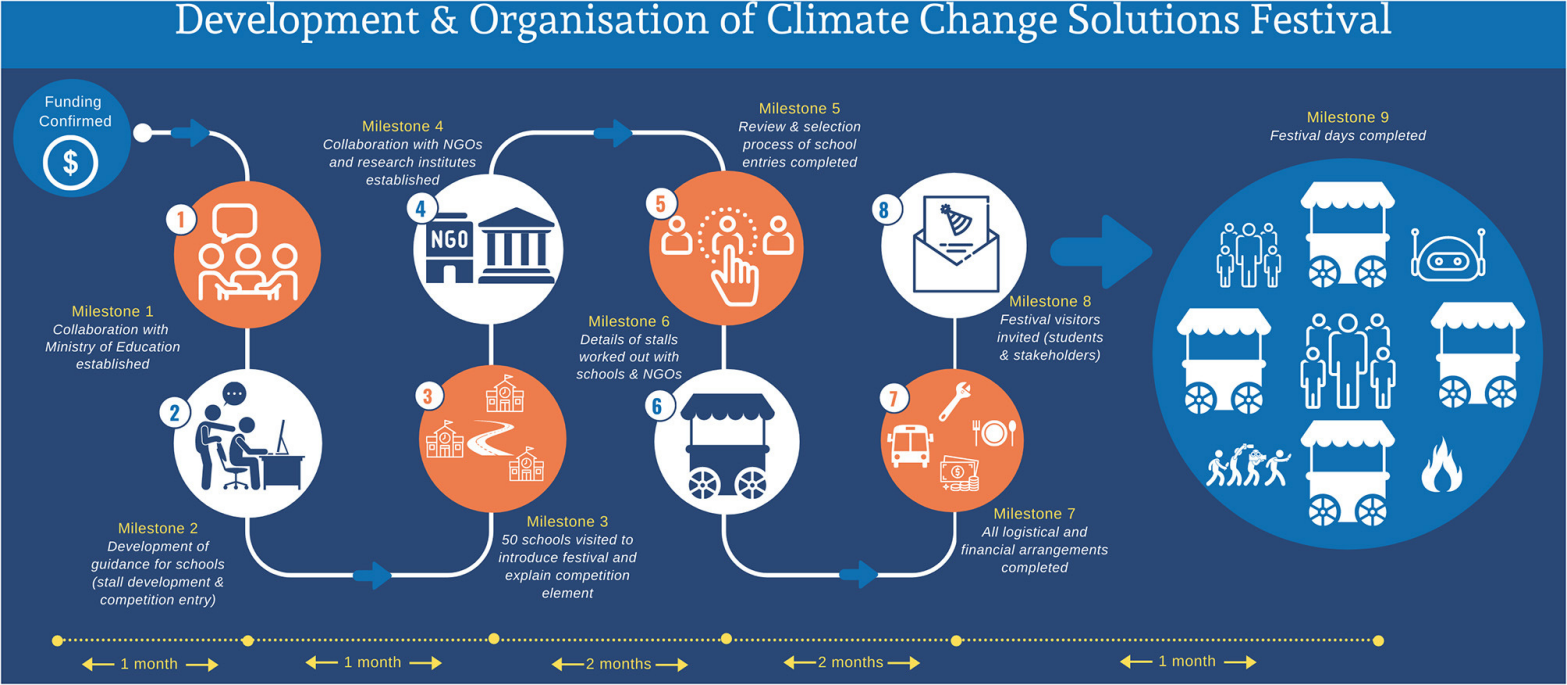
Timeline of organisational events leading up to the festival.

Conceptualization and initial planning for the Climate Solutions Festival built upon prior public engagement activities in The Gambia, the UK and India. Figure 1 gives the detailed timeline leading up to the festival. In brief, we received confirmation of funding in July 2020. In November 2020, in collaboration with the Ministry of Education (MoE) in The Gambia, we determined target year groups from Gambian schools across the country as well as local grass-roots NGOs that were active within The Gambia to invite to the festival to attend as students or demonstrators. We also determined which regions to approach based on logistical, time and cost constraints, received approval to visit the schools and discussed future work on expanding the coverage of climate change in the school curriculum. The festival's objectives were determined at this meeting with the MoE.

### Objectives

1. To enrich the debate around local climate change adaptation and mitigation by giving youth a voice/platform and encourage the dialogue between youth and decision-makers, NGOs and other stakeholders
2. To stimulate peer-to-peer teaching and learning among school-aged children
3. To develop open-source “*Climate change mitigation and adaptation options in the local environment*” resources—based on the science festival displays—with technical details for climate change projects suitable for use in schools and local communities.

We aimed to meet the above objectives by:

- Engaging 50 secondary schools to participate in a nationwide competition for grade 10-12 students (and their teachers)—aged 16-18 years and inviting them to submit ideas to interactively demonstrate climate or environmental change solutions.
- Selecting the most promising ideas for an interactive stall and offer demonstrators assistance (both financial and logistical) to develop their ideas.
- Inviting environmental, conservation and climate action groups active within The Gambia to develop and run interactive stalls on climate or environmental change solutions targeted to 13-16-year olds.

### Selection of Schools and Other Demonstrators

We included all regions in The Gambia except region three, which was logistically too challenging to visit (due to poor transport infrastructure). All eligible schools (for participation in the festival) required an in-person visit to explain in detail the festival aims: therefore, the maximum number of invitees was set to 50 to stay within time and budgetary constraints. Based on the total numbers of schools per region, we estimated the proportional number of schools to invite by region (out of 50) to observe diversity and regional representation. We communicated the total number of invitees per region to the regional education directors (members of the Ministry of Education overseeing schools in their region) and asked them to select specific schools for our invitational visits. Upon visiting the schools, the team explained the aims of the festival and delivered an invitation to participate in the competition as well as application forms.

A panel of five reviewers (AB, JB, ZA, KM, and PS) doubly assessed and scored all submissions based on three criteria: feasibility, level of interaction and visualization. To reduce regional bias and to allow voices from across the country to be included, schools were scored within each region and the number invited to participate was again proportional to the number of schools in the region. The top 11 schools were invited to demonstrate their ideas in a stall at the festival. Selected schools were supported financially and logistically and encouraged to make their stall highly interactive.

All environmental, conservation and climate action groups, known to the extended organizing team, and active within The Gambia were also invited to run interactive stalls at the festival and supported in the development of their ideas.

The final content areas (reflecting layout on the day) of the festival were divided into four main categories: trees and forests, food and agriculture, plastic and waste, and oceans and waterways.

### Winning Schools

**Figure 2:**
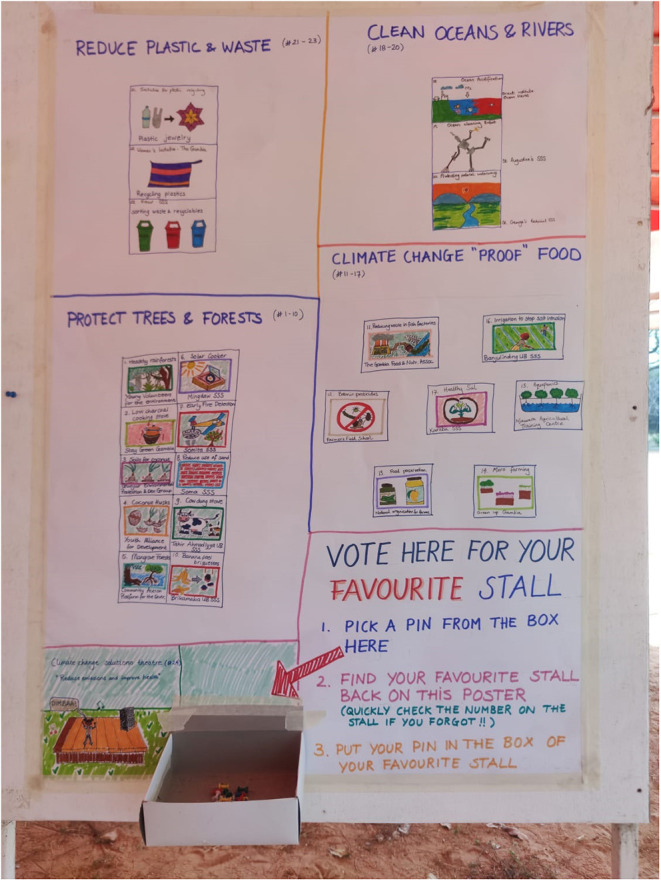
Student's voting board. Each student placed a pin in their favourite stall.

**Figure 3:**
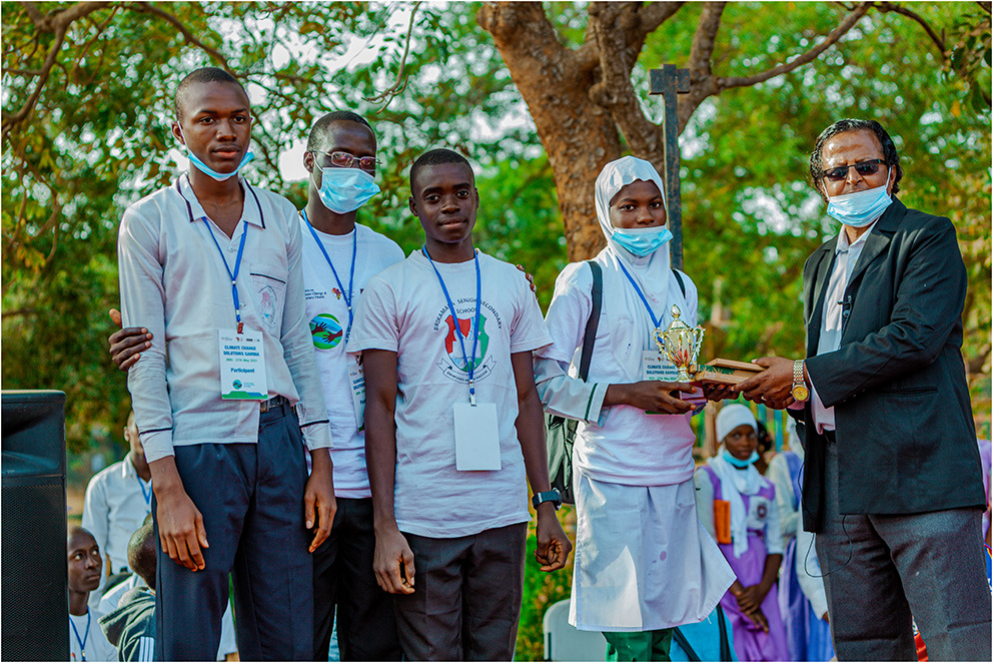
Demonstrators from the winning stall (banana charcoal) receiving prize from Mr Faal, Ministry of Education.

On the days of the festival, the school stalls were judged by an expert panel [comprising two senior scientists and the head of communications for the Medical Research Council Gambia (MRCG)] as well as student peers. Expert judges scored the stalls on: effectiveness of solution; visualization of the stall; and communication of the message. Peers voted on their favorite overall stall (Figure 2). The scores from peers and experts were combined to determine the overall “winners” of the festival (Figure 3).

### Observer Schools and Other Festival Participants

The primary audience of the festival were students in upper basic schools (aged 13-16 years). Six schools were invited from region one and transported to and from the festival grounds by the organizing committee. Key stakeholders from different governmental departments, NGOs and civil society organizations were also invited to observe and interact with presenting schools.

### Environmental and COVID-19 Considerations

To reduce environmental harm from the festival itself, we used compostable food trays, mainly vegetarian catering, water dispensers and plant-based biodegradable drinking cups. To protect against COVID-19 transmission, the event was held entirely outside with open-sided markees; all demonstrators and visitors were provided with surgical masks and encouraged to wear them throughout; hand-washing stations were positioned at regular intervals; and local transmission rates were deemed very low at the time.

### Ethics

Ethical approval was not sought for this initiative due to the nature of the festival and lack of data collection. Written informed consent was granted by legal guardians or individuals over 18 for display of any identifiable images.

### The Festival

**Figure 4:**
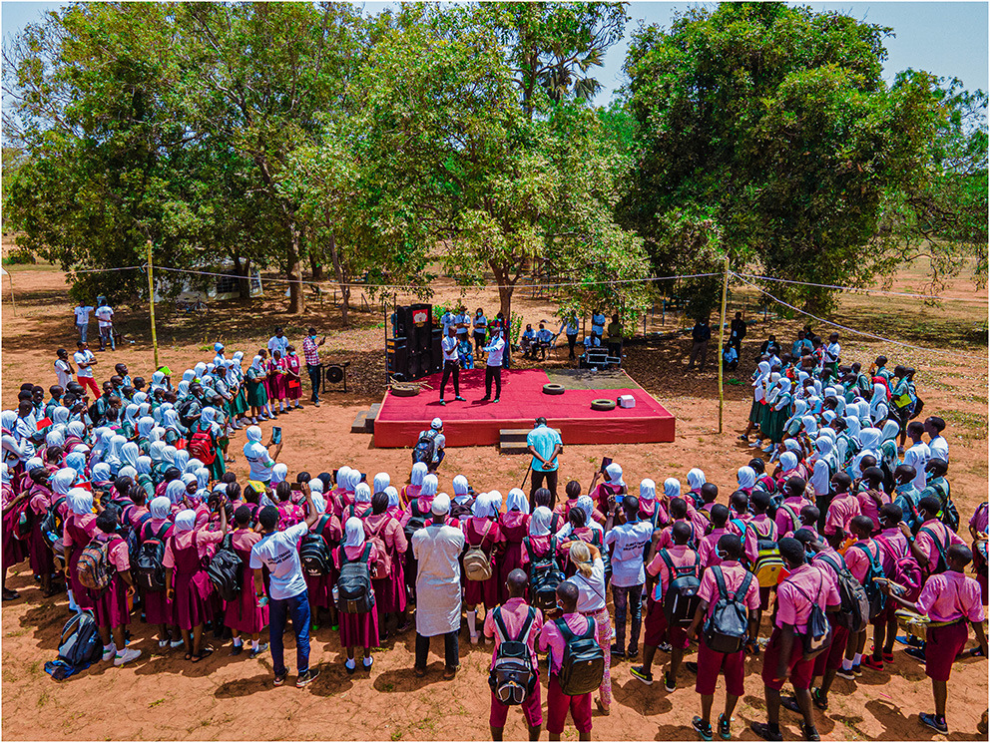
Festival attendees watching the theatrical performance on dangers of burning car tyres and benifits of alternative options.

The festival ran on 26th-27th May 2021 and involved 600 school children visiting and participating, with 200 in attendance at any given time (Figure 4). There were 21 stalls active during the festival (see Table 1 for the summary). Full details of all stalls can be found in the supplement—*Climate change mitigation and adaptation options in the local environment—*a toolkit for teachers, educators and students to recreate the experiments.

**Table 1:** List of all festival stalls.

**Name of NGO or school**	**Stall theme**	**Number**
**Trees and forests**		
Young Volunteers for The Environment	Solar cooker (protecting forests)	1
Gunjur Environmental Protection and Development Group	Reforestation and its many benefits	2
Youth Alliance for Development	Coconut husks as mulch and other nature-based solutions	3
Stay Green Gambia	Improved cook stove (mud stove)	4
Community Action Platform for the Environment	Importance of mangrove forests	5
Mingdaw Senior Secondary School	Solar Cooker	6
Somita Upper Basic and Senior Secondary School	Smoke detector for bush fires	7
Soma Upper Basic and Senior Secondary School	Air pollution and health/Alternative construction material	8
Tahir Ahmadiyya Upper Basic and Senior Secondary School	Cow dung oven	9
Brikamaba Upper Basic and Senior Secondary School	Banana charcoal briquettes	10
Kalagi Upper Basic and Senior Secondary School	Theatre—harmful practice of burning tires	21
**Food and Agriculture**		
Farmers Field School (Department of Agriculture)	Botanic pesticide/insecticide.	11
National coordinating organization for farmers association The Gambia	Food preservation	12
Kairaba Senior Secondary School	Cover cropping and organic manure	13
Banjulnding Upper Basic and Senior Secondary School	Salt intrusion	14
**Oceans and waterways**		
Great Institute (Ocean Heroes)	Ocean and estuary acidification	15
St. Augustine's Senior Secondary School	Plastic cleaning robot for the ocean	16
St. George's Technical Senior Secondary School	Protection of freshwater ponds	17
**Plastic and Waste**		
Plastic Recyclers Gunjur	Recycle plastic to make building tiles/bricks	18
The Women initiative The Gambia	Recycle plastic and tyres	19
Kaur Senior Secondary School	Importance of waste management	20

We present below a selection of the most popular stalls, highlighting the health and environmental benefits of these proposed solutions, as well as a narrative review of the global context for each topic.

### Alternative Fuel for Cooking

The most popular topic overall included solutions aimed at providing alternative fuels for cooking−5/21 of stalls focused on this. The demonstrators explained that these solutions address the combined impacts of air pollution on human health and biomass harvesting (e.g., firewood) from the environment. The benefits advocated included reduced respiratory diseases, reduced greenhouse gas (GHG) emissions and protection of forests (22–24). The following alternative fuel options were presented: (1) banana charcoal made of chopped banana peeling mixed with powdered grass/leaf charcoal and sand to from round briquettes; (2) clay cow dung oven made from clay collected at the river, shaped, dried and fired over 3 days and then dried animal dung used as fuel; (3) mud oven made from clay and sand, designed to improve cooking efficiency and so reduce fuel consumption; (4) two different solar cookers made from locally available materials in which the demonstrators cooked rice to indicate efficiency. Full details of how demonstrators made each of these alternatives are available in the supplement.

### Global Context

There are ~3 billion people globally that use biomass for cooking (25). The health effects of this results in an estimated 3.5 million deaths annually due to household air pollution (26), and there is a significant environmental cost to biodiversity due to deforestation driven by fuelwood production (27). In The Gambia, as in other SSA countries, firewood, charcoal and other biomass make up approximately 85% of local energy consumption (28). Burning biomass for cooking results in individuals exposed to harmful air pollutants, release of GHG emissions, deforestation and habitat degradation related to firewood collection (29).

Utilizing existing biomass waste (e.g., banana charcoal) have been minimally explored. A study by Mopoung and Udeye (30) found that banana peel briquettes were smokeless when burnt; however, combustion efficiency—a measure of how effectively the heat content of the fuel is transferred into usable heat—is low (9.1% compared to 80% for firewood) and therefore end-users may find it slow to cook food or heat water (36 vs. 18 min to reach maximum temperatures/boil water) (30, 31). However, overall, banana charcoal or utilizing biomass waste, with its simple and practical methodology should make it a priority to explore in future work as a potential locally realistic solution.

Despite the many benefits of alternative fuels, solar cookers and ICS, none of them have had extensive uptake globally (32, 33). A recent review of barriers to uptake of ICS identified 31 significant factors that determined whether an intervention to introduce ICS would be successful or not. Solar cooker uptake also suffers with similar problems (33). Identified barriers to use relate to issues such as perception of only targeting the poor, space and position limitations and cost, as well as the necessity to co-develop the end product with the end-users and ensure low or easy maintenance (34). Solar cookers should not be considered a purely low-income country alternative, as a study from Spain demonstrates potential annual life-cycle cost reduction of 40%, energy savings of 65% and electricity use reduced by 67 GWh/yr for solar cooker use compared to microwave use (35).

The clear message from our festival is that alternative fuel options are a priority for those living in The Gambia, highlighting a clear need to engage with communities on the issues around uptake of alternatives and to help identify effective solutions to reduce the amount of firewood and charcoal use.

### Solutions to Address the Impacts on Food Systems of Climate Change

#### Solutions for Saline Water Intrusion

Protecting food and agriculture from climate change impacts featured highly in the various types of solutions that were proposed by participating students. Where students from rural areas—further inland—were mainly focusing on alternative fuel and deforestation, students from schools closer to the coast aimed to address another climate change related impact on the food system: saline water intrusion and its negative impacts on crops.

The solution presented by the students aimed at building embankments around coastal agricultural land, diverting encroaching sea water into canals that were dug just outside the embankments. The students explained that they would use a fresh-water pond in the middle of the agricultural plot (inside the embankments) for irrigation with fresh water.

In addition, students explored the possibility of creating co-benefits of salt-intrusion by harvesting salt from salt evaporation ponds for later sale. The technique of raking salt from the bottom of salt evaporation ponds has existed for centuries and is a very effective, low-cost, traditional technique that could contribute to income diversification for farmers.

#### Global Context

Due to a number of gradual changes and shocks in climatic and environmental conditions, the frequency and intensity of saline water intrusion due to floods—especially in deltaic areas around the world—have been increasing (36). Weather events such as cyclones, tsunamis, extreme rainfall, extreme tides and sea level—all related to climate change—are important contributors to flood risks (37, 38). Saline water intrusion has been described in detail in South and South East Asia, including in the Ganges-Brahmaputra Delta (39), where it has an impact on drinking water quality and human health (40–42). However, inland encroachment of saline water is also experienced along the African coast (43) and can lead to substantial declines in crop yields if no appropriate adaptation measures are in place (44). The high salt concentration makes water uptake more difficult for the majority of crops, leading to reduced growth (45). Furthermore, excess sodium is toxic to several crops and crop plants, in particular, vegetables are highly salt-sensitive and salt-tolerant varieties are not as abundant as for the majority of staple crops (46, 47).

Worldwide, in the year 2000, ~620 million people lived in low-elevation coastal zones, of which ~93 million in low-income settings with often more limited possibilities to switch to salinity-tolerant varieties or diversification to other crops, livestock and/or alternative income sources (48). At the same time salinity problems are increasing, with salinity causing an estimated 50% reduction in global rice production (49). With still 6.7% (47 million) of children under five in the world severely underweight, many of them living in low-lying deltaic areas, it will be crucial to prevent a further decrease in food availability to ensure additional progress to SGD2: eradicate hunger. While the longer-term solutions are embedded in climate change mitigation strategies, in the immediate term, low-input solutions for saline water intrusion, that—preferably—can be developed and managed locally, are pivotal to successful adaptation and ensure food system resilience. Whilst the “diversion” of seawater is not widely practiced and would be technically challenging given the subterranean flows of saline water into soils and ponds (50), barriers to keep saline water at a distance from crop land are already widely used and show great potential as an adaptation strategy, provided numerous conceivable costs could be circumvented (51). Canal-type barriers filled with fresh water are used to create a “push-back” to the encroaching sea water, whilst various types of vegetation, including mangroves, salt marshes and seasonal grasses are often used as infiltration zone, before the seawater reaches agricultural land. A study from Khulna showed rice yield increased up to 41% in areas with mangrove planting vs. no flood protection (52). Salt mining would be an added co-benefit of managing saline water and help rural communities to diversify their income sources.

### Solutions for Food Preservation in Hotter Climates

A second food related topic covered at the festival addressed food preservation. The National Coordinating Organisation of Farmers Association (NACOFA) from Brikama demonstrated a number of food preservation techniques for a variety of foods. The organization explained that due to the increasing peak temperatures during the day preservation was pivotal in avoiding substantial amounts of food waste. In addition, given the increasing risk of yield failures, food preservation could be an effective solution to ensure food supply during the “hungry season” (wet season, July-Sep). Preservation techniques presented varied widely and included: (1) sugar-preservation techniques, such as preparing kabaa jam (*Saba senegalensis*) by boiling and jarring kabaa fruit with sugar and lime; (2) salting techniques, also applied to fruits like the *Saba senegalensis*, with added pepper, salt and flavor enhancers; (3) drying/dehydration techniques, for example applied to “*mbahal*” (rice, dried fish *(kobo)* and flavor enhancers) or “*findi”* (also called *Fonio*—a type of millet).

### Global Context

Food preservation techniques date back to as far as centuries ago—with use of salt as preservation technique known to have been used in Egypt as early as 2000BC (53). However, with increasing discovery and use of preservatives in food and introduction of (affordable) refrigerators for domestic use, the need for preservation at household level changed drastically. Nonetheless, climate change has posed a renewed necessity for preservation in some areas, especially in areas experiencing prolonged periods of extreme heat. It started to be mentioned as a climate change adaptation strategy in the food and agricultural sector 10-15 years ago [e.g., (54)], and has been slowly coming back into mainstream practices, especially in the Global South. Parajuli et al. (55) highlight that supply of (especially perishable) produce is highly dependent on logistical management, transportation and refrigeration and/or preservation requirements. As a consequence, food waste is directly linked to the application of food preservation and refrigeration. Several case studies from small island states also illustrate that (traditional) food preservation techniques could increase food security in the aftermath of natural disasters (56).

### Solutions to Reduce Plastic Pollution in the Environment

While the link between climate change and plastic pollution might not be well known (see global context below), there was high interest in the issue of environmental plastic pollution among students: 2/11 of all solutions presented by the participating schools focused on plastic. Two schools—focusing on plastic pollution in oceans and in the urban environment, respectively, were shortlisted for demonstration at the festival.

The school aiming to reduce plastic in oceans designed, engineered and demonstrated a floating solar powered plastic cleaning “robot,” made primarily from waste materials, complete with a moving conveyer belt to transport floating plastic from the surface of water bodies into a container with a capacity of around 20 L, as well as rudders to passively guide flotation. The robot was demonstrated to effectively capture polystyrene pieces floating on the surface of a paddling pool.

### Global Context

While the link is often not well-known to the wider public, plastic in the environment certainly contributes considerably to climate change. Plastics in the environment slowly release greenhouse gases (mostly methane and ethylene) throughout their lifetime (57), especially when exposed to substantial amounts of heat and sunlight (58). Furthermore, presence of (micro)plastics in the ocean will substantially interfere with carbon fixation (57). Shen et al. estimated that greenhouse gas emissions from all plastics (cradle to grave) will reach 1.34 Gigatonnes per year by 2030, seriously affecting carbon budgets and the ability to stay below +1.5°C above pre-industrial levels (57). Ocean plastics contribute to this problem: annual methane production of the standing stock of ocean pollution is 76Mt of methane, which translates into ~2.1 Megatons of CO_2_ (59). The robot design demonstrated was not unlike commercially available plastic traps in current use or trials (60), for examples the Seabin and FRED (Floating Robot for Eliminating Debris) (61, 62). The makers of Seabin estimated that each 20 L bin is able to capture 1.4 tons of debris per year, while the current network of 860 bins worldwide captures more than 3.5 tons per day (~1,200 tons per year) and more than 2,000 tons have been collected since their deployment (61, 62). However, capacity needs to be substantially increased to meet current rates of plastic pollution, which have been estimated to average 8 million tons per year with a standing stock of >200 million tons, and is increasing at an alarming rate (63). Given the rapid increase in use of plastics, and the fact it also hinders the carbon sequestering capacity of oceans, its future impact on climate change is projected to become more dominant.

## Discussion

Substantial negative impacts of climate change on the lives of many residing in West Africa is a daily reality and is of particular concern for the youth residing in the region. As “experts by experience,” there are often many practical ideas and solutions among members of the public on how to adapt to or mitigate these impacts, but not all voices have a platform.

The Climate Change Solutions Festival allowed students to demonstrate their ideas, interact with educators, researchers, NGO workers, decision-makers, funders and other stakeholders and engage in peer-to-peer teaching and learning related to practical knowledge and skills on climate change mitigation and adaptation options in their local environments.

Student engagement and stakeholder-student interaction give a platform for young people to share their concerns and ideas on climate change. As future leaders on climate change action their early engagement in research and decision-making in climate change is crucial in two ways: their concerns—and also possible solutions to overcome them—may give better insight in climate change impact at the grass-roots level, while their engagement in the topic at a younger age will make them (and their generational peers) more aware, knowledgeable, enthusiastic and equipped to work toward climate change mitigation targets in the future.

### Peer Learning

Peer-to-peer teaching and learning has been used for decades to increase student motivation, responsibility, commitment, and sense of purpose while using various learning styles that appeal to their peers (64). In a global climate crisis that will affect many generations to come such elements are crucial for effective future leadership in climate change action. In addition, the group work element of the presenting teams at the festival allowed them to negotiate what problems to tackle, what solutions to present and what mode to use for their communication to ensure comprehension for their peers (65). Hence, there were many opportunities to strengthen transferrable skill, such as listening, explaining, questioning, summarizing, speculating, and hypothesizing (66). In the predominantly teacher-oriented pedagogic styles adhered to in many low-income settings (67), including the Gambia, the trusting relationship between peers (whereby no one holds a position of authority), may facilitate self-disclosure of ignorance and misconception, which will likely accelerate learning (66). Students appeared to appreciate the often enthusiastic, competent and sometimes humorous presentations of the stall-members. However, we did not directly evaluate this or ask for feedback from the students on the format of the festival, rather we asked more generic questions regarding learning on the day and overall enjoyment. In future endeavors it would be helpful to thoroughly evaluate this style of learning.

### Co-creation of Research and Policy by Citizens and Practice Communities

The student-stakeholder interaction created a platform for practice communities with young people at its center, to voice their experiences and ideas for improvement and therewith potentially influence decision-making in practice and policy. The crucial importance of practice communities and citizens in climate change action and decision-making is increasingly acknowledged by national and international governing and advisory bodies. The Danish government, for instance, co-created their national climate strategy with members of the public (Climate Consortium Denmark), while the UK Government organized a citizen's Assembly on Climate Change to explore: “*How should the UK meet its target of net zero greenhouse gas emissions by 2050?”* (68). With several government officials and other-decision makers present during our festival, mainly due to the support from the Ministry of Education but also through long ties with our Institution and many key decision makers reflecting a long history of public health research and collaboration, this could accelerate the process of citizen's involvement in The Gambia, or other West African contexts. Despite this, we did not evaluate communications between students, stakeholders and NGOs to assess whether our objective of encouraging dialogue was met. Moreover, in order to foster transformative change there needs to be ongoing projects, communications and interactions which the festival organizers did not actively seek out and set up.

All proposed solutions have their highly context-specific benefits, some more than others, backed up with scientific evidence. Their presentation feeds back into research in two ways: (1) the problems addressed shed light on perceived problems and local priorities and hence could direct research focus in the local area, and (2) the solutions posed could be further refined and tested and their benefit for health and the environment could be calculated for use in cost-benefit analyses. Additionally, to act upon these proposed solutions in the community would require close collaboration with local authorities and communities to assess feasibility to the wider population of The Gambia.

### A Positive Note in the Climate Crisis

The applied nature of the solutions posed by students and NGOs and the interactive components that allowed participants to explore local solutions to climate change adaptation and mitigation in great depth would likely have contributed community building and knowledge sharing. Some of the problems addressed at the festival, such as deforestation, increased heat and water salinization are well documented, but community members often feel powerless or out-of-depth in their personal attempts to take action. The practical solutions aiming to reduce or prevent climate change impacts may have formed a positive message and a start for finding scalable solutions that resonate with the users/implementers. The data captured during the festival in personal conversations, feedback forms, photos, notes from the festival stalls, and in-person evaluations with organizers are shared and presented in this manuscript as well as in a practical booklet describing the climate change mitigation and adaptation options. (*Climate change mitigation and adaptation options in the local environment—Climate Change Solutions Festival Banjul—May 2021.)* This could be used as a “handbook” for education on local climate change action, as well as a starting point for community involvement in developing future climate change strategies.

## Methodological Constraints

While we intended to get wide-spread representation of students and NGOs from various areas in The Gambia, logistical constraints prevented all schools from being able to participate, further highlighting the widespread problem of underservice in remote communities (25). In addition to this the six schools that attended the festival were all from region 1—the region where the capital is situated and therefore the area with many existing opportunities. Future programs or festivals must consider equity of access, inclusivity and fairness going forward.

In future we would also have defined evaluations of the designated objectives to allow a thorough assessment of successes and areas in need of improvement.

## Data Availability Statement

The original contributions presented in the study are included in the article/Supplementary Material, further inquiries can be directed to the corresponding author/s.

## Ethics Statement

Written informed consent was obtained from the minor(s)' legal guardian/next of kin for the publication of any potentially identifiable images or data included in this article.

## Author Contributions

AB and PS conceived, organized, enacted project and wrote first draft. JB coordinated festival and edited manuscript. SJ, ZA, AA, KM, and AP were on the organizing committee and edited manuscript. MH, AD, MF, and WH involved in organizing festival, demonstrated on the day, and edited manuscript. All authors contributed to the article and approved the submitted version.

## Funding

Funding was secured from LSHTM small grant continued development grant with additional funding from Wellcome Trust through the FACE-Africa project (grant no. 216021/Z/19/Z) under the Wellcome Climate Change and Health Award Scheme. AB was funded by a Wellcome Trust Global Health PhD Fellowship (216336/Z/19/Z).

## Conflict of Interest

The authors declare that the research was conducted in the absence of any commercial or financial relationships that could be construed as a potential conflict of interest.

## Publisher's Note

All claims expressed in this article are solely those of the authors and do not necessarily represent those of their affiliated organizations, or those of the publisher, the editors and the reviewers. Any product that may be evaluated in this article, or claim that may be made by its manufacturer, is not guaranteed or endorsed by the publisher.
