# Supplementary material for: Grassroots and Youth-Led Climate Solutions From The Gambia
Source: Front Public Health. 2022 Apr 7;10:784915. doi: 10.3389/fpubh.2022.784915 (PMC9021377; doi:10.3389/fpubh.2022.784915)
Supplement: Supplementary file 1 [file Data_Sheet_1.pdf]

Climate change mitigation and adaptation options in the local environment

Climate Change Solutions Festival

May 2021

This learning and action pack details the stalls presented at the Climate Change Solutions Festival in Fajara, The Gambia, West Africa, May 2021.

We would like to take this opportunity to acknowledge all the schools that participated in both the competition, demonstrating and attending. Both the teachers and students were fantastically engaged in developing the stalls and in the peer-to-peer learning that occurred during the festival.

Additional thanks goes to all the other stall demonstrators who dedicated their time to improving the understanding and discussions around climate change solutions and helped inspire the attendees.

Contents:

Stall Theme	Number
**Trees and Forests**	
Solar cooker (protecting forests)	1
Reforestation and its many benefits	2
Coconut husks as mulch and other nature-based solutions	3
Improved cook stove (mud stove)	4
Importance of mangrove forests	5
Solar Cooker	6
Smoke detector for bush fires	7
Air pollution and health/Alternative construction material	8
Cow dung oven	9
Banana charcoal briquettes	10
Theatre	21
**Food and Agriculture**	
Botanic pesticide/insecticide	11
Food preservation	12
Cover cropping and organic manure	13
Salt intrusion	14
**Oceans and waterways**	
Ocean and estuary acidification	15
Plastic cleaning robot for the ocean	16
Protection of freshwater ponds	17
**Plastic and Waste**	
Recycle plastic to make building tiles/bricks	18
Recycle plastic and tyres	19
Importance of waste management	20

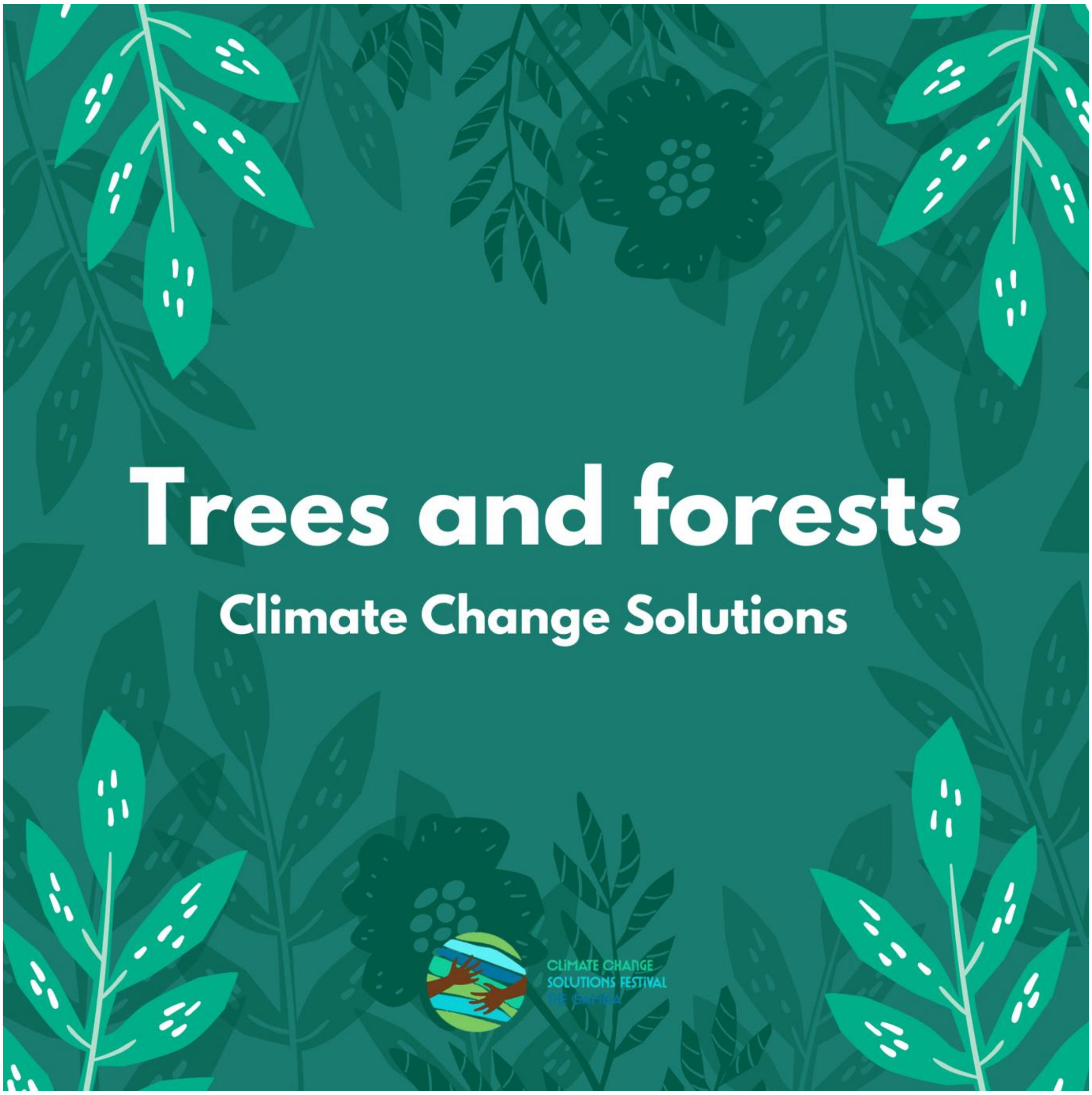
Trees and forests
Climate Change Solutions
CLIMATE CHANGE
SOLUTIONS FESTIVAL

1. Solar cooker

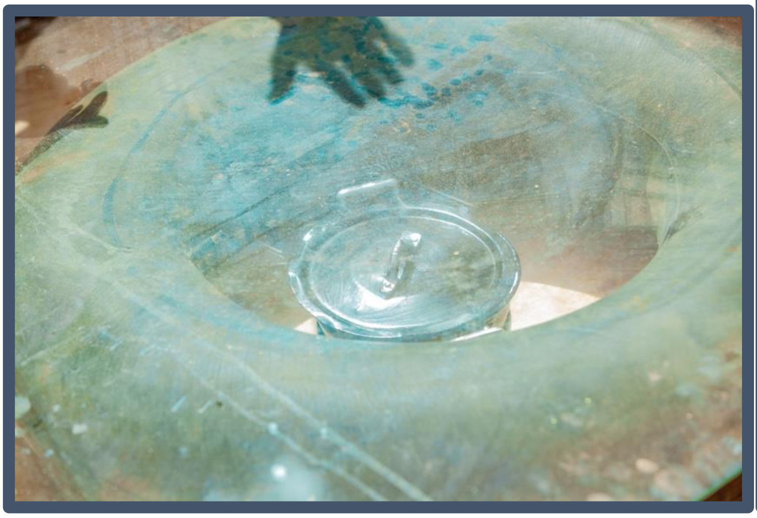
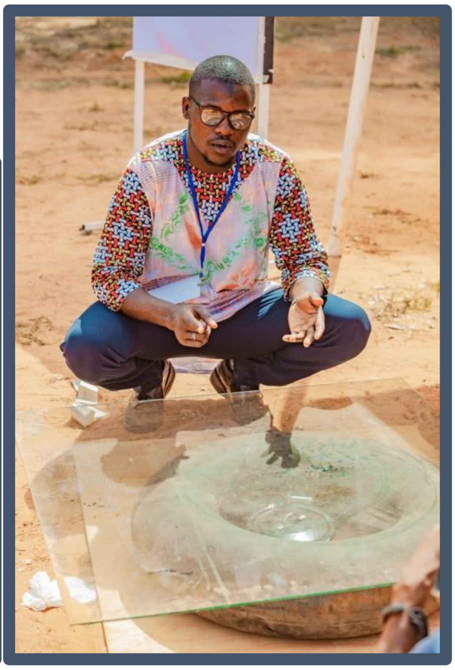

Basic principles: Reduce use of firewood for cooking

Interaction: build your own solar cooker using recycled items.

Equipment required:
Carboard
Inner tube of tire
Cooking pot
Glass

Cook with solar to reduce firewood and charcoal use

- Place a flat piece of cardboard or wood on the ground.
- Inflate the inner tube of a large car tire.
- Place your item to cook in the middle of the tire as shown in the picture.
- Place a large sheet of glass over the top and leave in the sun.

Experiment with boiling water and cooking rice – timing each to see how long they take to cook.
Explore solar cooker recipes: https://solarcooking.fandom.com/wiki/Recipes

2. Reforestation and its many benefits

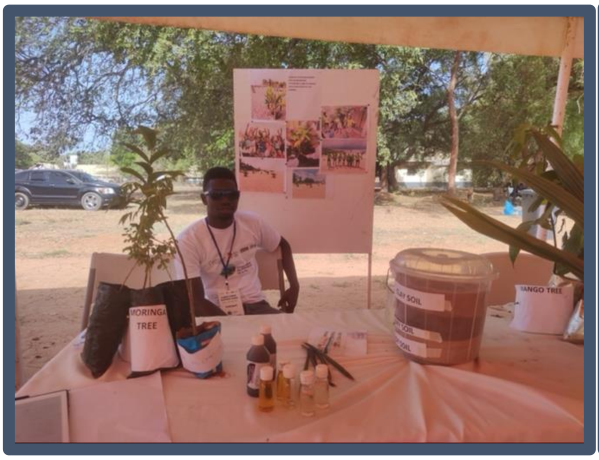

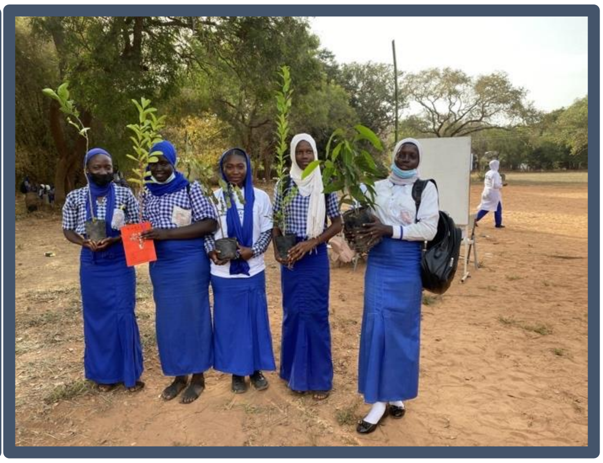

Basic principles: reforestation to improve the health of humans and the planet

Interaction: Explore different growing potential of different soil types

Equipment required:
Seeds
Soil
Plant containers
Bucket

Plant trees to protect the planet

Experiment: test which soils results in healthy seedlings

- Discuss benefit of trees generally (e.g. clean the air we breathe, clean the water we drink, provide shade, improve soil, reduce rainwater run-off, improve biodiversity and social/psychological benefits).
- Discuss specific considerations of coastal regions (e.g. sand encroachment, storm and flood protection etc).
- Experiment with growth of different trees in different soil types.
- Take a selection of seeds (moringa, oranges and mangoes) and take three different soil types: sandy, loamy and clay.
- Plant each seed in the three different soils and watch to see which one germinates and thrives.
- Will take several weeks.

3. Coconut husks as mulch/growing medium and other nature-based solutions

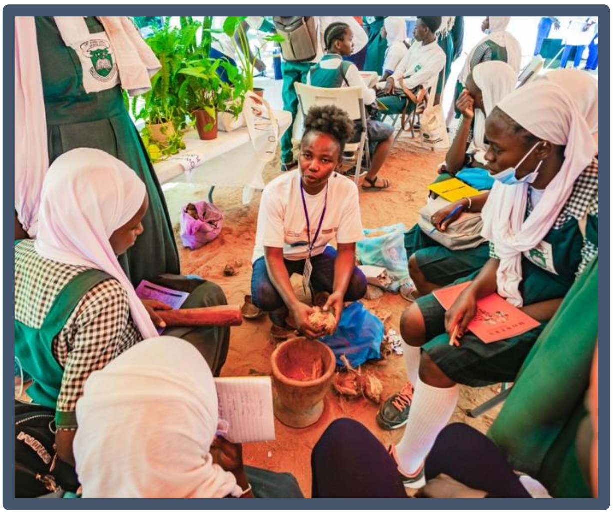

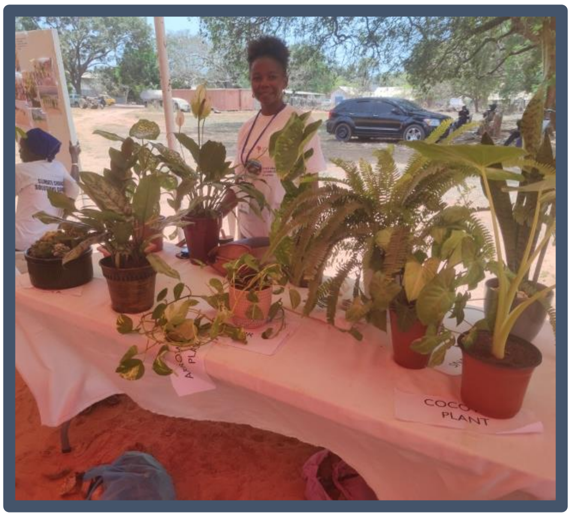

Basic principles: the importance of plants for a healthy planet & healthy humans

Interaction: making coconut mulch and biodegradable pots

Equipment required:

Pestle and mortar — Scissors
Coconut husks (dried) — Paper tape
Newspapers — Glass jar

More plants = cleaner air

- Discuss the benefits of using coconut husk as mulch/growing medium (e.g., water retention ability, aeration capacity, sustainable, naturally pH balanced, nutrient density, beneficial bacteria and cost free) and
- Allow students to pound coconut husk to powder.
- Discussed on the benefits of houseplants (e.g. cleaner indoor air).
- Explain benefit of biodegradable pot versus plastic for planting
- Made paper pots:
- Take one sheet of newspaper
- Place glass jar one third from the top and at the horizontal edge and then roll with the newspaper.
- Tape the cylinder then remove the glass jar.
- Fold the bottom and tape closed to give a paper pot.
- Add soil and coconut mulch and seed/seedling. Can be planted straight into the ground.

4. improved cook stove (mud stove)

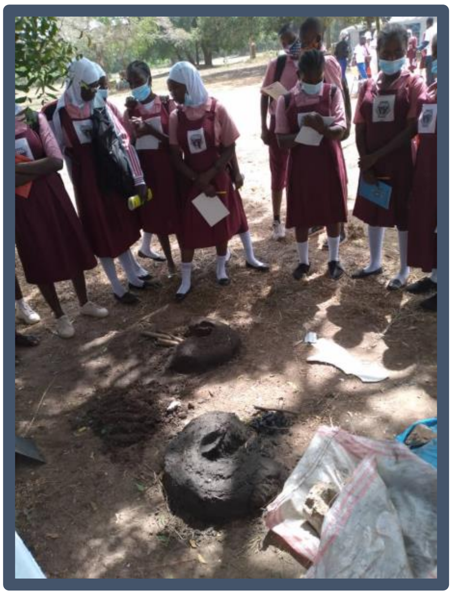
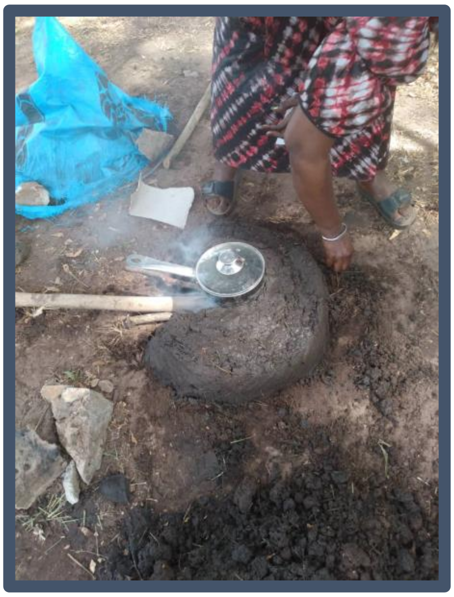

Basic principles: improve efficiency of stoves to reduce firewood/charcoal use

Interaction: Build your own mud stove.

Equipment required:
Bucket
Straw
Clay soil
Firewood
Cooking pot

Efficient cook stoves save trees

Experiment: test efficiency of mud stove

- Collect large bucket of clay soil.
- Collect one bucket of straw.
- Use the outer edge of your cooking pot to measure out an arc and place the straw in this position on the ground.
- Add water to the clay soil slowly and mix until it becomes muddy.
- Shape the mud into rectangular blocks and place on top of the straw, three blocks high.
- Leave to dry for one day.
- Use firewood in the mud stove and place the cooking pot on top. Test length of time to heat water compared to conventional stove.

5. Importance of mangrove forests

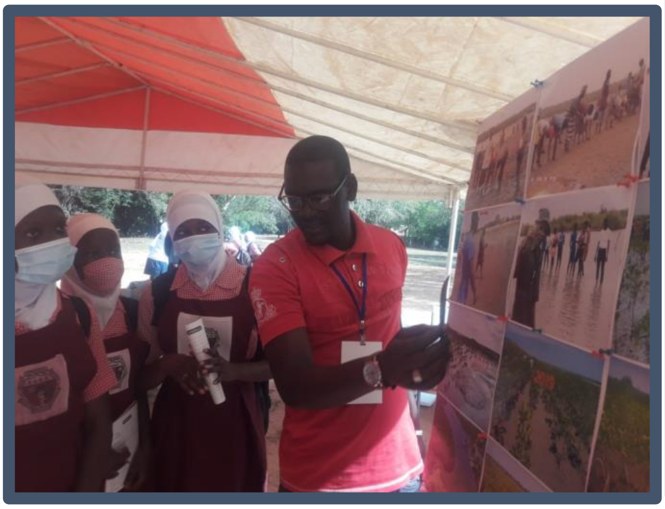
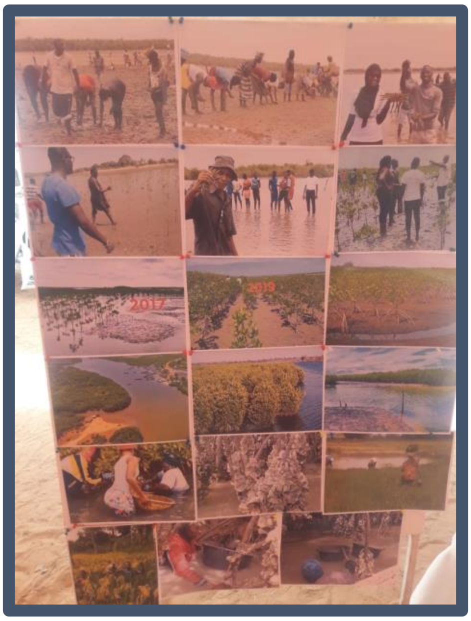

Basic principles: costal protection & costal ecosystems need healthy mangroves

Interaction: discuss importance of mangroves & practice planting mangrove seedlings

Equipment required (for in class presentation):
Clear photos of planting mangrove forest
Clear photos of the protective effect of mangrove forests
Mangrove shoots & seedlings

Planting mangrove forests protects the environment

Experiment: demonstrate the multiple benefits from mangrove forest planting

- Develop a clear presentation board indicating 1) every step of mangrove forest planting in the community; 2) several examples of the protective effect of mangrove forests.
- Explain the main steps of mangrove forest planting, including the direction (in rows) and the way how seedlings are planted
- Show seedlings and show how they are planted: this can be practiced by the participants
- Explain benefits of mangrove forests, including 1) possibilities to harvest oysters that grow on mangrove roots; 2) their positive impact on biodiversity, by creating favourable habitats for birds and bees; 3)highly salt absorbing: lowering salinity in ground and surface water that is used for irrigation; 4)

6. Solar cooker

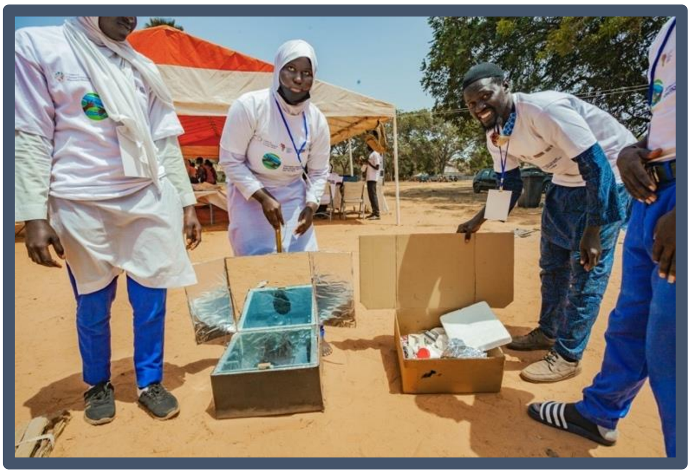

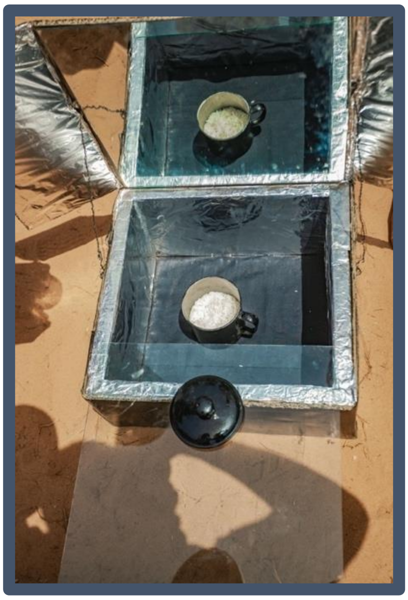

Basic principles: Reduce use of firewood for cooking

Interaction: make your own solar cooker

Equipment required:
Carboard box
Polystyrene
Black paint
Aluminium foil
Cooking pot
Glass sheet

Solar cookers are great for The Gambia

Discuss important factors for a solar cooker: time of day for cooking, need to reflect and focus sunlight, need to insulate – give the students time to come up with ideas on how to maximise each of these components.

To make the solar cooker:

- Take one carboard box. Paint the outside of the box with black paint.
- Line the inside of the box with polystyrene and cover with aluminium foil as shown in the picture. Cover the inside of the lid with aluminium foil or a mirror if available.
- Paint the cooking pot with black paint and allow to dry
- Experiment with cooking different food and drink.

7. Smoke detector for bush fires

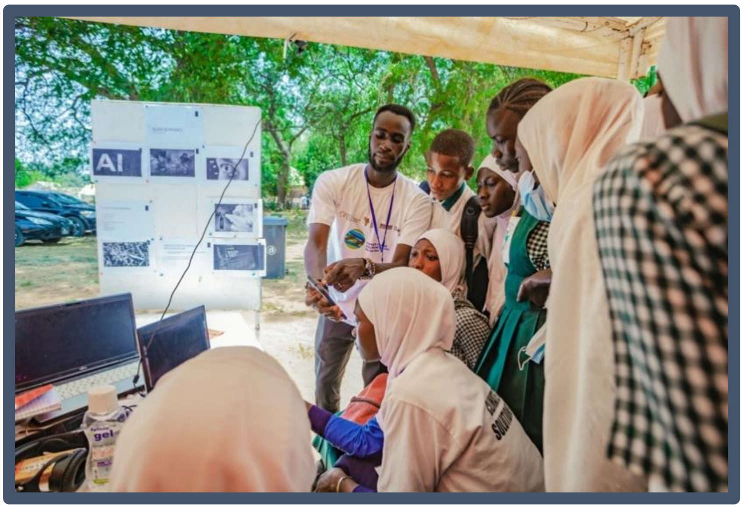

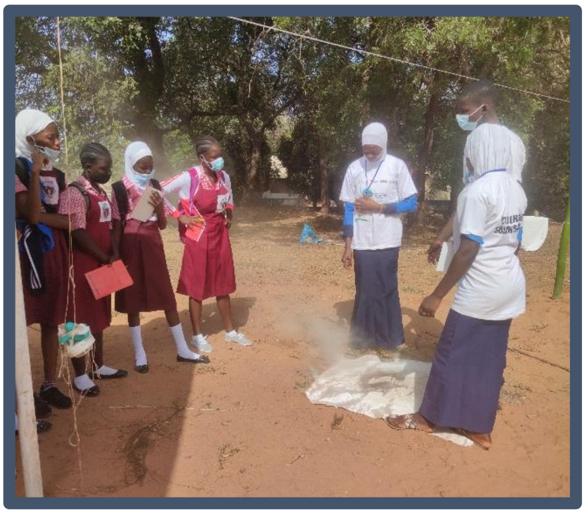

Basic principles: Reduce the damage of bushfires

Interaction: early recognition of bushfires to reduce damage

Equipment required:

**Smoke detector**	**Wood/dry grass**
**String**	**Fire blanket/extinguisher**
**Model aeroplane**	**Matches**

Bushfires harm life

Discuss the damage of bushfires and impact on human health (air pollution). Demonstrate the use of a smoke detector with smart phone connection for early recognition and intervention.

Set-up: make a fire pit in a safe place. Put wood, kindling and dry grass ready for ignition. Use string to suspend the smoke detector attached to the model aeroplane (as a surrogate for a drone) over the area of the fire pit. Ensure the smartphone app is installed to allow communication between the smoke detector and the phone.

- Divide students into 3 groups and separate where they cannot see each other.
- Group 1 starts the fire and ensures it does not spread.
- Group 2 has the smartphone and receives the signal to indicate there is a fire and informs group 3.
- Group 3 run to extinguish the fire (with fire blanket/extinguisher).
- Can be timed and run as a competition to see who can extinguish the fire the quickest.

8. Health impacts of air pollution from cars & alternatives to sand for construction

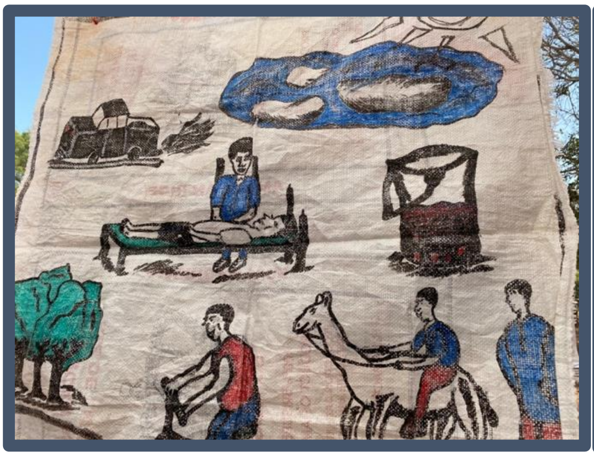

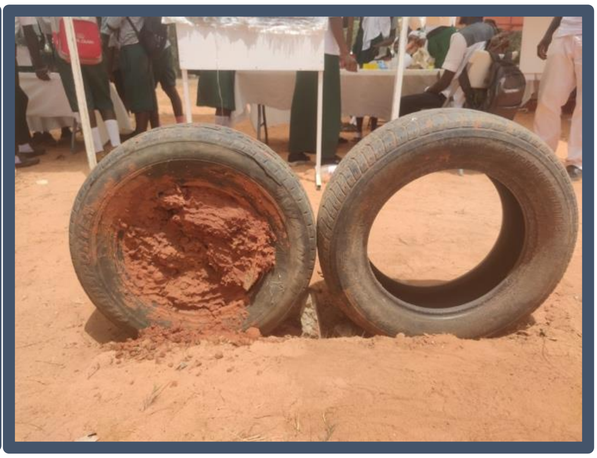

Basic principles: Solutions to reduce air pollution

Interaction: Health impacts of air pollution and potential solutions

Equipment required:
Paper
Paint
Pens

We want clean air

Experiment:

- Discuss health impacts of air pollution (asthma/respiratory disease, cardiovascular disease etc).
- Give the students an opportunity to discuss causes of air pollution and potential ways to reduce this (including clean cars/legislation regarding fuel type/use of trees and plants to clean the air etc).
- Allow students to draw their own posters to spread awareness.

Basic principles: To provide a clean alternative for housing construction

Interaction: Experiment with alternative construction material for sustainable living

Equipment required:
Tyres
Stones, Quarry Dust
Sand and Mud

No to erosion

Experiment:

- Discuss problems with sand mining (erosion/loss of biodiversity etc.) and discuss impact on livelihoods & environment
- Allow students to consider alternative options and share experiences with alternative building materials
- Encourage students to experiment and build model houses out of their proposed material and test for strength of building blocks.

9. Clay cow dung oven

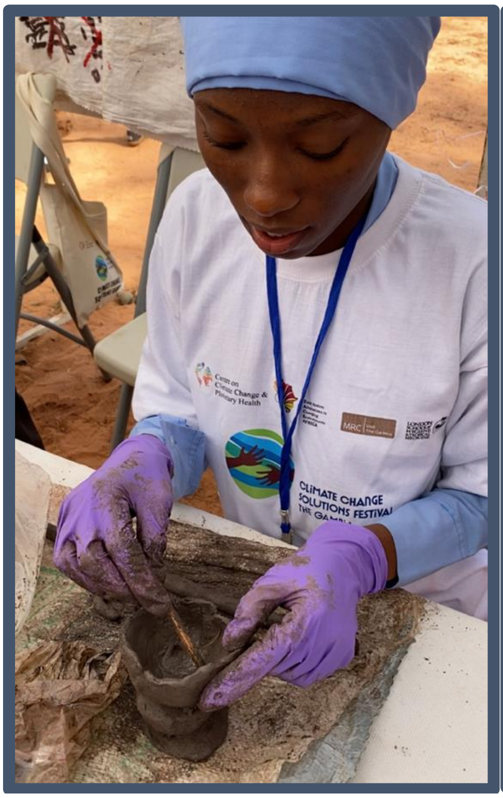
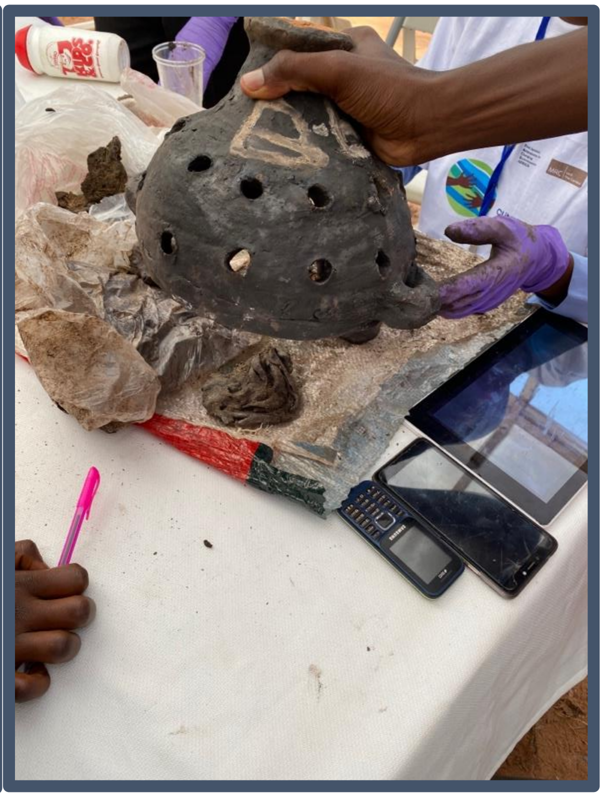

Basic principles: Reduce use of firewood for cooking

Interaction: build your own clay oven

Equipment required:
Clay
Sticks
Dry grass
Cow dung

Don't plant back trees – just don't cut them!

Experiment

- Collect clay from the river.
- Leave to dry in the sun. Alternative option is to recycle old clay objects – pound and sieve and mix together.
- Allow students to design their own ovens similar to the pictures shown.
- Leave to dry for 3 days.
- Fire with cow dung and dry grass for 6-7 hours.

Once cooled experiment with cooking different foods. Fuel = cow dung.

10. Topic: Banana charcoal briquettes

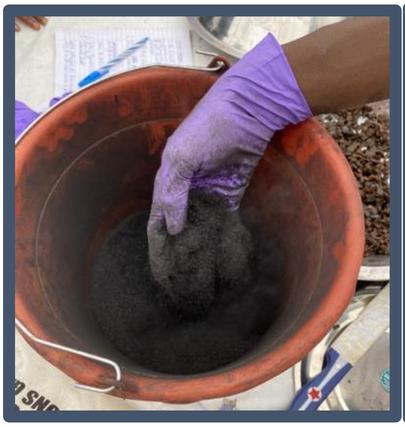

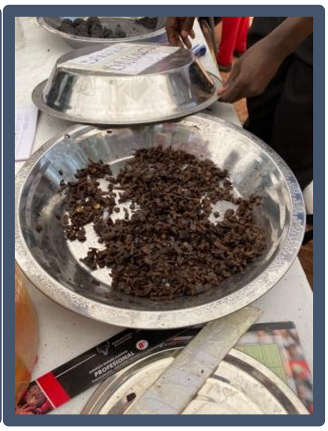

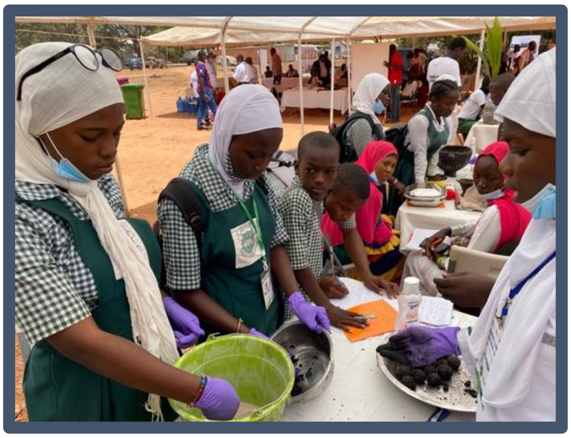

Basic principles: Reduce use of firewood for cooking

Interaction: make banana charcoal briquettes.

Equipment required:
Grass
Leave fall
Banana peelings
Sand
Sieve
Knife

Burns as well as other charcoal but produces no smoke and doesn't chop down a single tree.

- Collect dried grass/leaves and slow burn under a mound of earth with minimal oxygen to produce charcoal.
- Collect, pound and sieve the charcoal to give charcoal dust (see picture).
- Collect fresh banana peelings and cut into small pieces (see picture).
- Mix banana peelings (50%), charcoal dust (25%) and sand (25%) together and mould into small briquettes.
- Leave to dry (from 2-4 days depending on season). Burns with minimal smoke.

Test against other charcoal for time to boil a set volume of water.
Additional experiment: make charcoal briquette from other household organic waste and test heat efficiencies as above.

Food and farming

Climate Change Solutions

CLIMATE CHANGE SOLUTIONS FESTIVAL THE GAMBIA

11. Botanical pesticide/insecticide

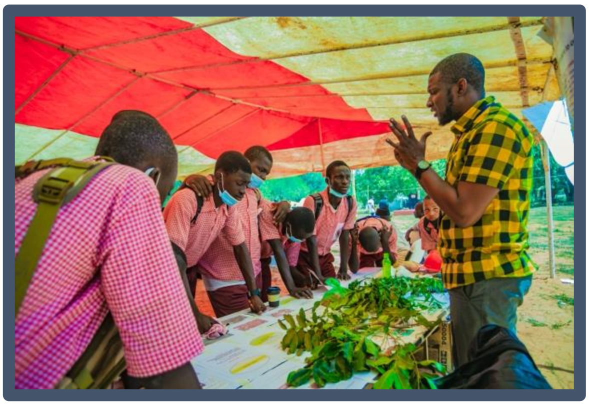

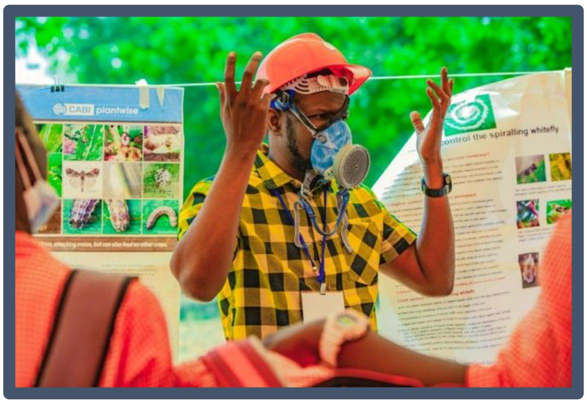

Basic principles: An environmentally friendly alternative to chemical pesticides

Interaction: Seeing and touching the plants, Wearing protective equipment for chemical insecticide application

Equipment required:
Plants to make botanical pesticides/insecticide
Spraying cans
10 litre barrel/bucket with water
Sieve
Mashing pestle

Botanical pesticides
are good for you,
the environment
& our crops

Experiment:

- Brief explanation of the various plants that can be used as botanic pesticide and insecticide, including leaves of eucalypt, African mahogany and neem trees
- Use a pestle to crush the plant leaves (and in some cases seeds)
- When using leaves: add half of a bucket of leave to a full (10 litre) bucket of water. Add lid and leave to brew for a few days. For attendees on the day, take a mixture that has already brewed for a few days.
- Sieve the mixture and fill spraying cans
- Show pictures of experiments with crops/trees (e.g mango & orange) with and without botanical pesticides/insecticides and show the difference in yields

Furthermore, participants can try on the protective equipment for the application of chemical fertilizer (full body suit, boots, google) to show the challenges of use of such equipment in hot climates.

12. Food preservation

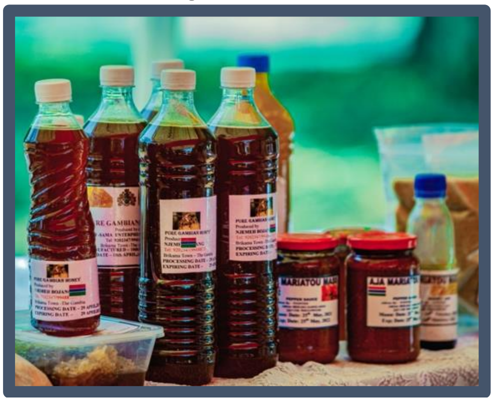

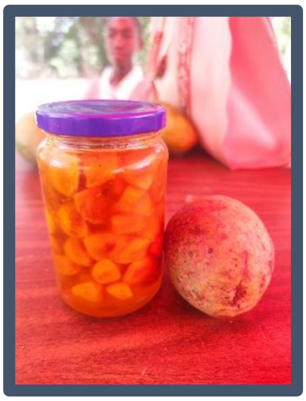

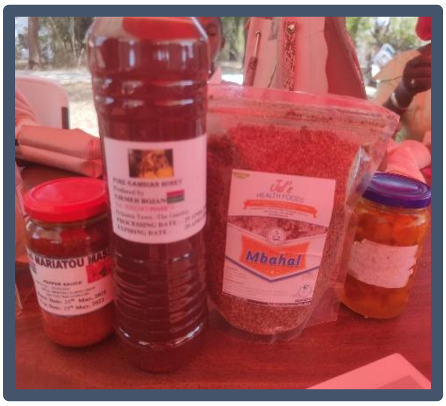

Basic principles: Prolong the shelf life of perishable products through various preservation techniques

Interaction: Tasting foods, trying out preservation technologies

Equipment required:
Bottles and jars
Plastic sealable bags
Stove
Pot
Sugar, lime, salt, pepper, other preservation ingredients

No food waste with these preservation techniques

Experiment:

- Explain that due to the increasing peak temperatures during the day preservation is important to reduce food waste.
- Explain that food preservation could be an effective solution to ensure food supply during the hunger season.
- Demonstrate the preparation of kabaa jam (Saba senegalensis) by boiling kabaa fruit with sugar and lime
- Demonstrate salting technique of the Saba senegalensis, with added pepper, salt and flavour enhancers
- Demonstrate drying/dehydration techniques of "*mbahal*" (rice, dried fish *(kobo)* and flavour enhancers) or *"findi"*.
- Show participants the finished products that have been prepared before the demonstration
- Participants can practice the techniques with small amounts of food
- Participants can taste the different foods by providing small samples

13. Cover cropping and organic manure

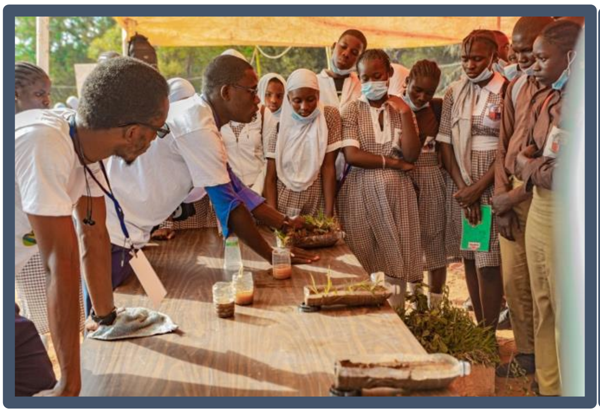

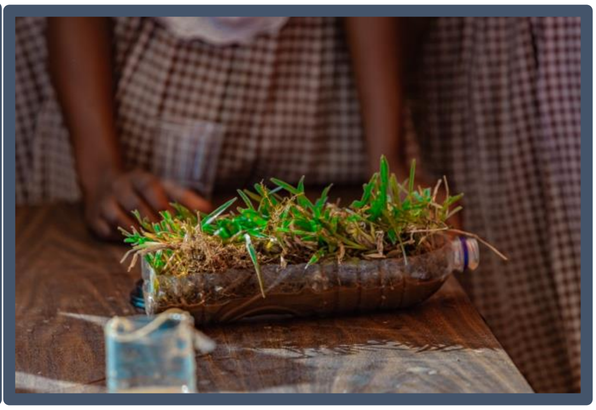

Basic principles: importance of healthy soil structure

Interaction: Demonstrate different growing potentials in different conditions

Equipment required:
Small plot of land
Soil
Animal dung
Seeds

Healthy soil = healthy plants

- Prepare 3 beds for planting
- All beds will grow corn and be watered with the same amount of water.
- Bed 1: plant corn and grow in soil with only watering.
- Bed 2: prepare soil with dried groundnut shells. Plant corn. Water as needed.
- Bed 3: prepare soil with manure. Plant corn. Water as needed
- Students are responsible for watering and weeding as needed.
- Around 3 months assess height of plants, width of stem, size and number of corn, and most importantly the taste of the corn.

14. Reducing salt intrusion

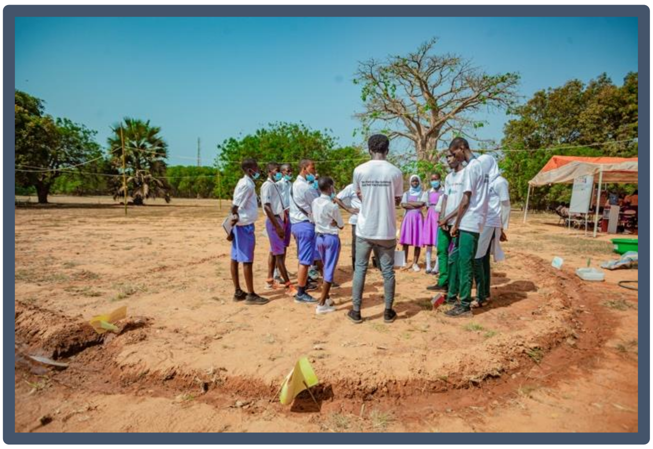

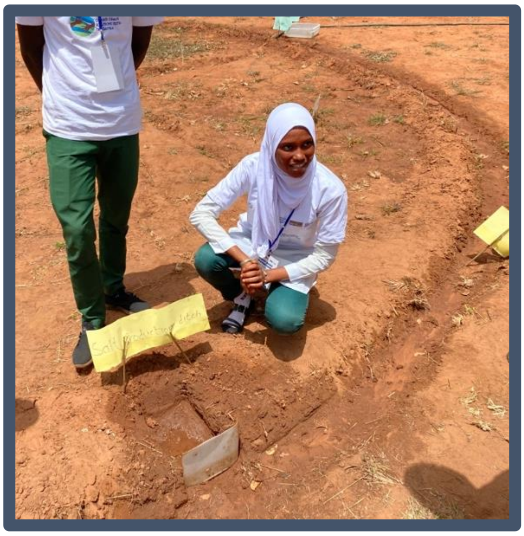

Basic principles: protection of land from salt intrusion

Interaction: build your own channels to divert salt water and create an artificial salt pan

Equipment required:
Land
Salt
Water
Spade
Bucket
Board (plastic/wood)

Saltwater intrusion will reduce our crops – let's find a solution

Discuss saltwater intrusion: principles of salt entering the ground and impact of climate change on this. Discuss impacts of salt water on soil fertility, crops growing potential and on human/animal health. Discuss ways in which this may be addressed and options – allow the students to generate their own thoughts and ideas.

- Dig channels as indicated in the picture on the left
- Dig a smooth flat area to make the artificial salt pan (picture on right)
- Use a large amount of salt water to fill the channels
- Open the board/barrier to the salt pan to allow the water in and then close off
- Leave the salt water to evaporate or stay in the channels

CLIMATE CHANGE SOLUTIONS FESTIVAL THE GAMBIA

OCEANS & WATERWAYS

CLIMATE CHANGE SOLUTIONS

15. Understanding ocean and estuary acidification

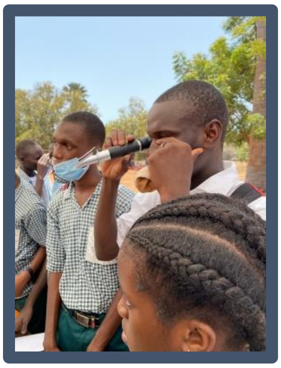 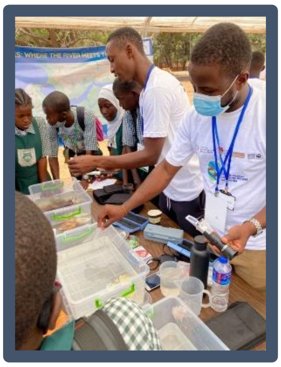

Basic principles: How climate change affects estuaries and causes ocean's acidification

Interaction: understanding marine ecosystems

Equipment Required:
pH meter
Dissolved oxygen meter (DO)
Refractometer
2 - 500 mL beakers
3 reusable plastic tanks
3 bamboo or paper straws
Materials to resemble a marine environment (coral, fish, shells, etc).

Ocean acidification harms our estuaries

Explain to the students what an estuary is. Discuss how carbon dioxide is released into the atmosphere then dissolves into the ocean causing an increase in the acidity of the water. Discuss the threat of climate change in the marine ecosystems.

To demonstrate an estuary:

- Collect or make freshwater, brackish water or saltwater and place each one of the reusable plastic tanks.
- Divide students into three groups. Each group will represent a fresh aquatic system, salt aquatic system, and a brackish aquatic system.
- Using the map of the River Gambia, demonstrate the areas of saltwater, brackish water, and freshwater.
- Using a refractometer, ask the students to determine which reusable plastic tank represents fresh, brackish or saltwater.
- Then use the dissolved oxygen meter to measure the DO of each aquatic system. Explain to the student why DO is important in an aquatic system.

To demonstrate ocean acidification:

- Collect or make 800 mL of saltwater and divide the sample into the two (2) 500 mL beakers.
- Ask students to blow air through the straw into each beaker containing the saltwater for three minutes.
- Using the pH meter, ask the students to measure the pH levels for both samples.
- Repeat steps 2 and 3 but ask the students to blow for six (6) minutes to measure the pH levels.
- Using the DO meter, repeat steps 2 and 4 to measure DO.

16. Plastic cleaning robot for the ocean

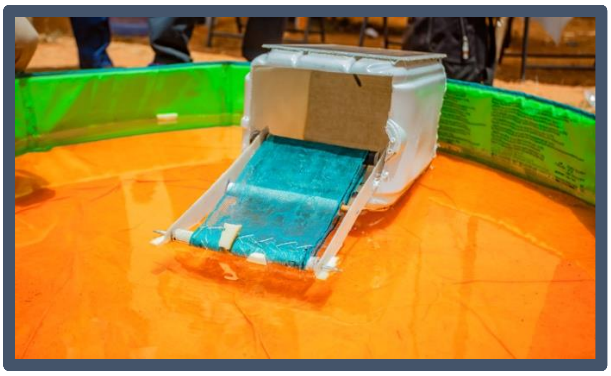

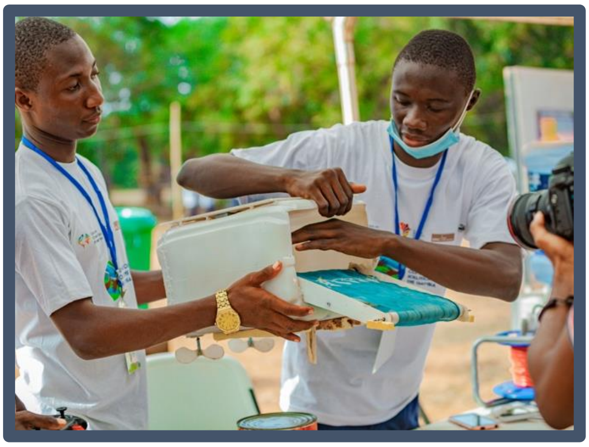

Basic principles: remove harmful floating plastic items from waterways

Interaction: highlight the impact of plastic use and build your own plastic cleaning robot

Equipment required:
Polystyrene box
Solar panel
Net
Small pieces of plastic rubbish
Dynamo
Small pool

Keep the ocean clean

Discuss ways in which we use plastic in our lives. Ask students to count the number of plastic items they use in one day. Ask students to think about what happens to plastic once it has been thrown away (e.g. length of time for degradation of 1 plastic bag = 1000 years). Most plastic ends up in landfill or water. The ocean now has 5.25 trillion pieces of plastic in it and that number is increasing daily.
What does the plastic in the ocean do?
What ways can the students suggest to clean plastic from the ocean?
Use equipment above to build their own robot similar to what is seen in the pictures.

17. Protection of freshwater ponds

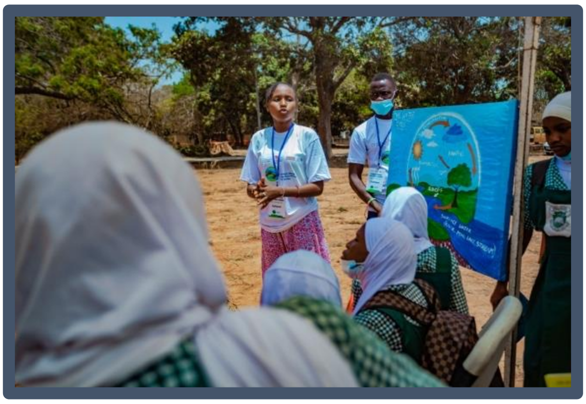

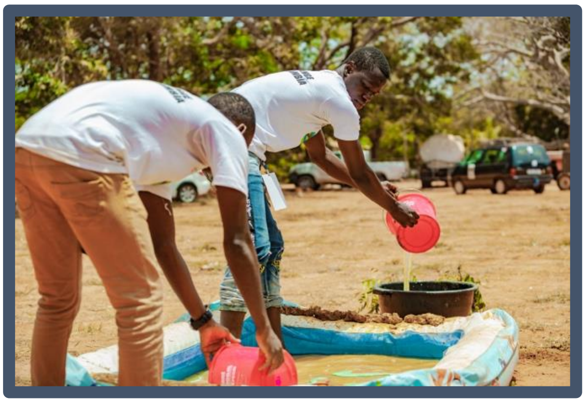

Basic principles: Importance of the water cycle and healthy freshwater ecosystems

Interaction:
Interaction: Use role play/theatrics to explore the stressors on the water cycle

Equipment required:
Paper
Pens
Art supplies

Plan for the future and care for our ponds. Don't drain them as once they are gone, they are gone

This may take several sessions.

1. Discuss the water cycle with the students and ask them to produce drawings to illustrate it.
2. Form small study groups and give each student the opportunity to present their drawing to the members of their group.
3. In the study groups discuss the water cycle, how humans may affect it and what impact that can have.
4. As a group, choose one impact that is the most important to them and develop a play to demonstrate/discuss this with potential solutions.
5. Each group performs their play to the whole group.
6. Finish with any final take home messages or questions from the groups.

CLIMATE CHANGE
SOLUTIONS FESTIVAL
THE GAMBIA

Plastics and waste

Climate change solutions

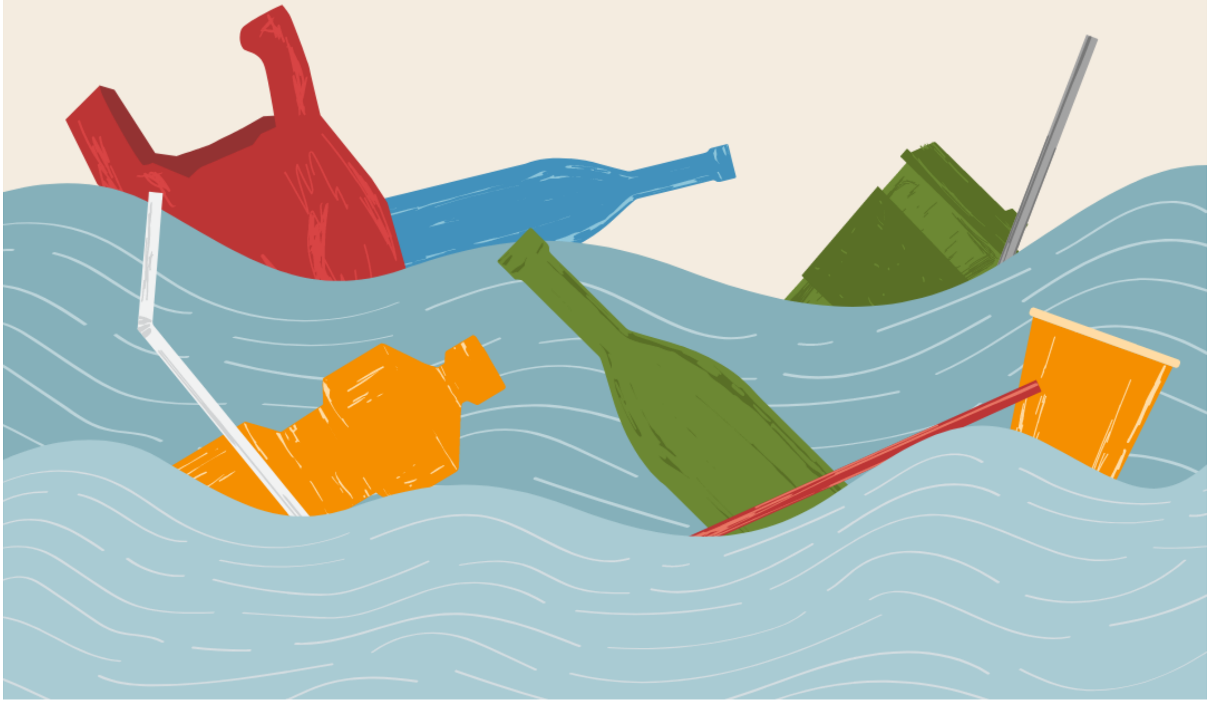

18. Recycle plastic to make building tiles/bricks

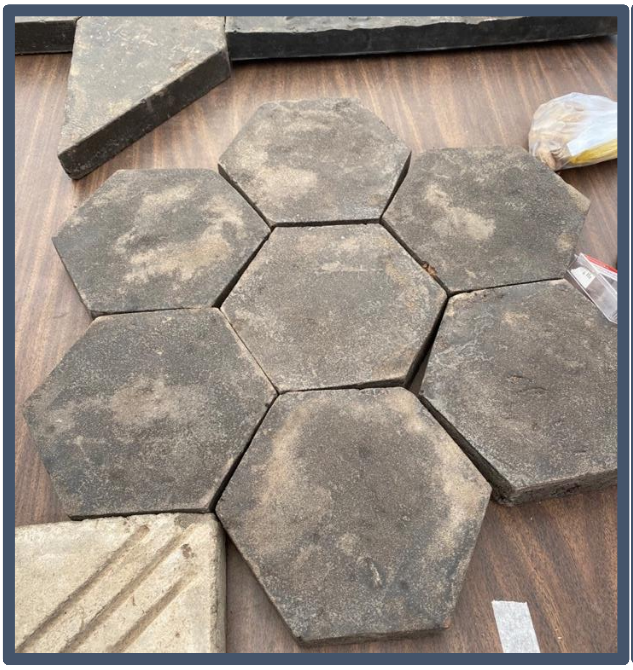

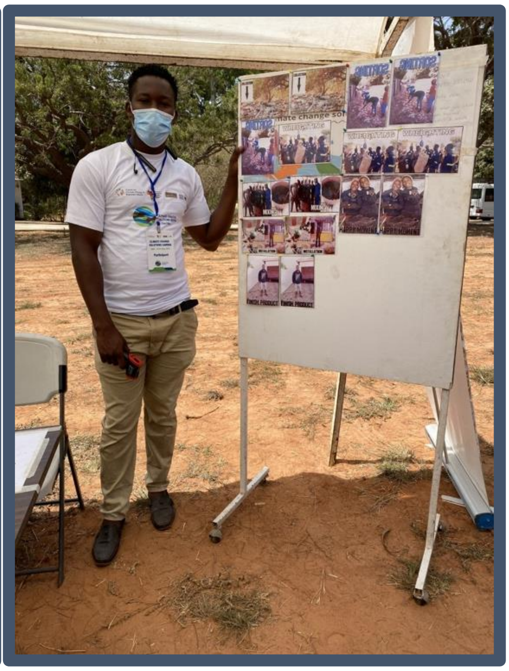

Basic principles: recycle low-density polyethelyne (LDPE) plastic to create tiles and bricks

Interaction: discuss plastic waste in The Gambia. Ask students if they think plastic is a problem and what can be done about it.
Explain the options for recycling plastics into other useful items – e.g. bricks.
Ask each student to collect any stretchy type of plastic they use over the next 2 weeks.
If feasible arrange a visit to Gunjur plastic initiative to give the plastic waste for recycling and see the whole process in action.

Details of the process of making plastic tiles:

- Collect plastic
- Clean and sort
- Use LDPE plastic
- Weigh total
- Melt plastic at 185°C
- Add sand
- Mix
- Pour into mould
- Leave for 15 mins then remove and ready for use

Build your home with recycled plastic

19. Recycling plastic and tyres

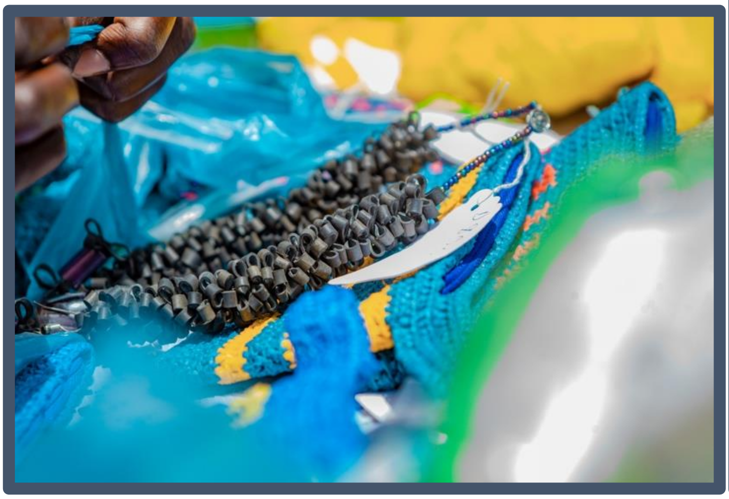

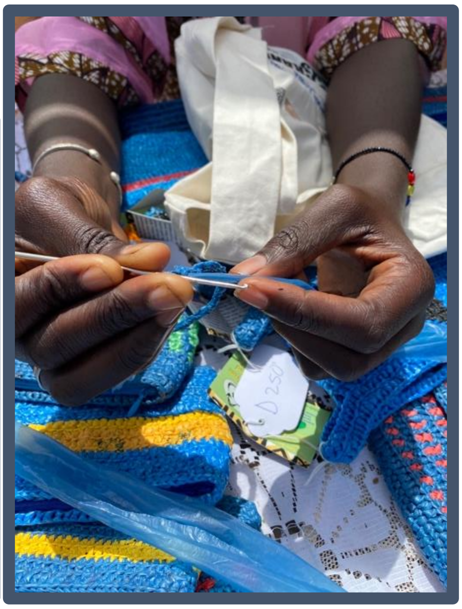

Basic principles: recycle plastic bags and tyres to make bags, purses and bowels

Interaction: make your own recycled purse/bag

Equipment required:
Bucket and cleaning product
Discarded plastic bags
Scissors
Crochet needle

There's too much plastic, so let's recycle

Discuss plastic waste globally and in The Gambia. Ask the students if they think recycling is important and why.
Ask students to collect discarded plastic bags.

- Clean the bags
- Dry them
- Cut into strips
- Crochet into bag or other item they prefer as seen in the pictures

20. Importance of waste management

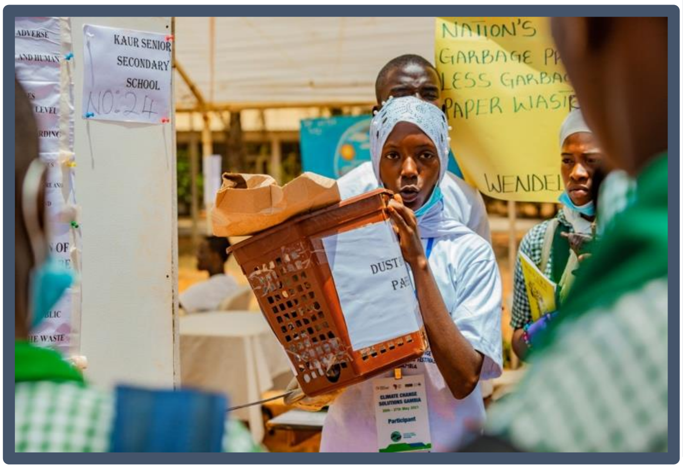

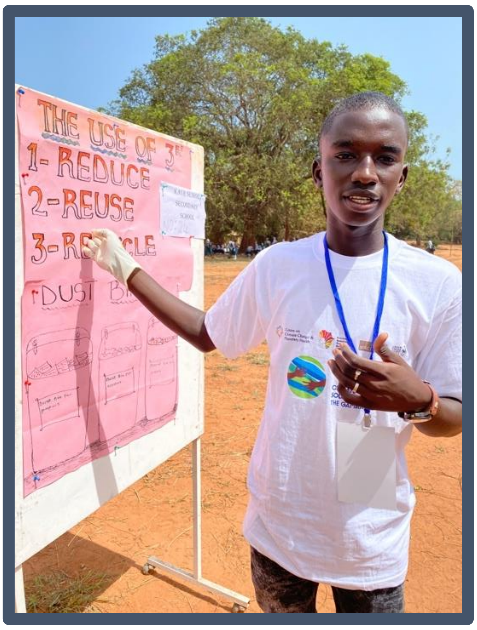

Basic principles: REDUCE | REUSE | RECYCLE

Interaction: waste sorting and reusing

Equipment required:
Household waste
Glue
Paint
Paintbrushes
Bins to sort rubbish into

Reduce
Reuse
Recycle

This will take place over 3 sessions.

1. discuss the principles of reduce, reuse and recycle. Consider waste management in their local area and identify if there is a problem – do the students know of any local actions to reduce waste?
2. Each student collects household waste (non-food waste only) for 1 week and then brings to school. Hold a competition for fastest and most accurate sorting of waste (e.g. cans/paper/plastic/glass).
3. Spend time using the household waste to create either something of use, or of artistic value.

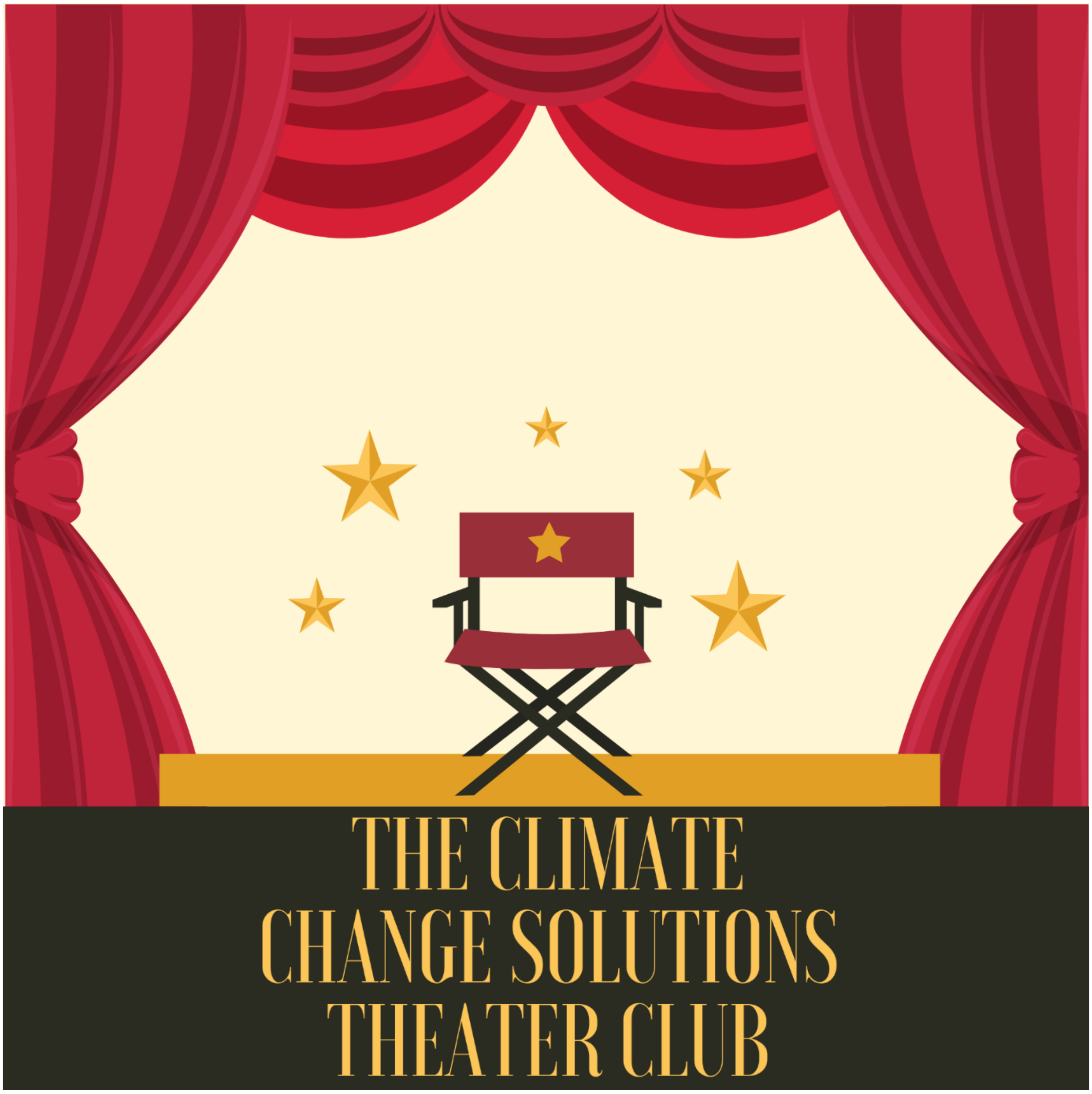
THE CLIMATE
CHANGE SOLUTIONS
THEATER CLUB

21. Climate change solutions theatre

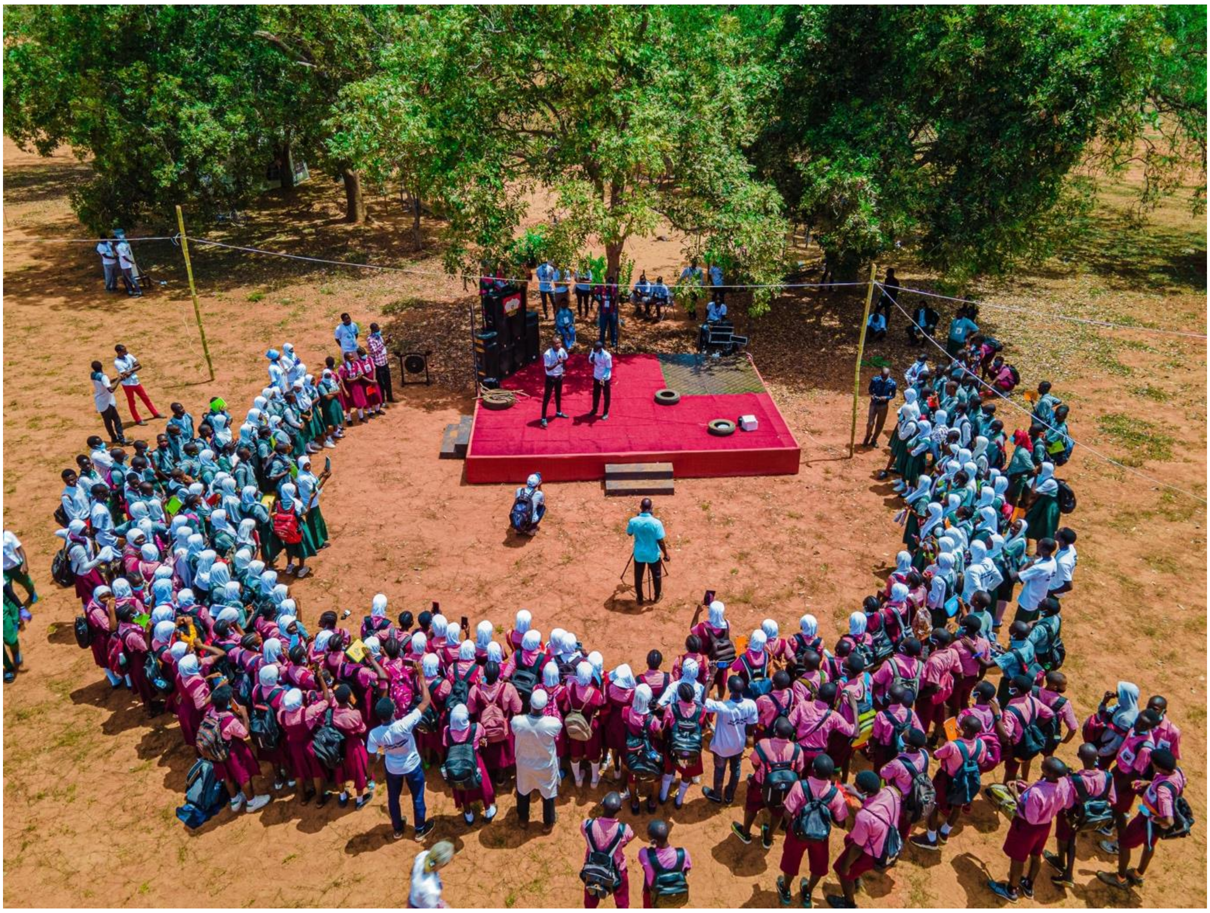

Basic principles: show a detrimental impact to health of climate change or environmental degradation

Interaction: Put on a play to the school

Learn through theatre

Equipment required:
Costumes
Other props as needed
Microphones

- Spend some time discussing different aspects of climate change and how it can impact human health
- Allow the students to form small groups and work on their ideas for a play
- Give them the opportunity to practice together
- Put a show on for the school to spread awareness
